# Bulk and single‐cell alternative splicing analyses reveal roles of TRA2B in myogenic differentiation

**DOI:** 10.1111/cpr.13545

**Published:** 2023-09-13

**Authors:** Genghua Chen, Jiahui Chen, Lin Qi, Yunqian Yin, Zetong Lin, Huaqiang Wen, Shuai Zhang, Chuanyun Xiao, Semiu Folaniyi Bello, Xiquan Zhang, Qinghua Nie, Wen Luo

**Affiliations:** ^1^ College of Animal Science South China Agricultural University Guangzhou Guangdong China; ^2^ Guangdong Provincial Key Lab of Agro‐Animal Genomics and Molecular Breeding, Lingnan Guangdong Laboratory of Modern Agriculture & State Key Laboratory for Conservation and Utilization of Subtropical Agro‐Bioresources, Key Laboratory of Chicken Genetics, Breeding and Reproduction, Ministry of Agriculture Guangzhou Guangdong China; ^3^ State Key Laboratory of Livestock and Poultry Breeding, and Lingnan Guangdong Laboratory of Agriculture South China Agricultural University Guangzhou China; ^4^ Human and Animal Physiology Wageningen University Wageningen The Netherlands

## Abstract

Alternative splicing (AS) disruption has been linked to disorders of muscle development, as well as muscular atrophy. However, the precise changes in AS patterns that occur during myogenesis are not well understood. Here, we employed isoform long‐reads RNA‐seq (Iso‐seq) and single‐cell RNA‐seq (scRNA‐seq) to investigate the AS landscape during myogenesis. Our Iso‐seq data identified 61,146 full‐length isoforms representing 11,682 expressed genes, of which over 52% were novel. We identified 38,022 AS events, with most of these events altering coding sequences and exhibiting stage‐specific splicing patterns. We identified AS dynamics in different types of muscle cells through scRNA‐seq analysis, revealing genes essential for the contractile muscle system and cytoskeleton that undergo differential splicing across cell types. Gene‐splicing analysis demonstrated that AS acts as a regulator, independent of changes in overall gene expression. Two isoforms of splicing factor *TRA2B* play distinct roles in myogenic differentiation by triggering AS of *TGFBR2* to regulate canonical TGF‐β signalling cascades differently. Our study provides a valuable transcriptome resource for myogenesis and reveals the complexity of AS and its regulation during myogenesis.

## Graphical abstract

Alternative splicing (AS) disruption has been linked to disorders of muscle development, as well as muscular atrophy. However, the precise changes in AS patterns that occur during myogenesis are not well understood. We employed isoform long‐reads RNA‐seq (Iso‐seq) and single‐cell RNA‐seq (scRNA‐seq) to investigate the AS landscape during myogenesis. Our Iso‐seq data identified 61,146 full‐length isoforms representing 11,682 expressed genes, of which over 52% were novel. We identified 38,022 AS events, with most of these events altering coding sequences and exhibiting stage‐specific splicing patterns. We identified AS dynamics in different types of muscle cells through scRNA‐seq analysis, revealing genes essential for the contractile muscle system and cytoskeleton that undergo differential splicing across cell types. Gene‐splicing analysis demonstrated that AS acts as a regulator, independent of changes in overall gene expression. Two isoforms of splicing factor *TRA2B* play distinct roles in myogenic differentiation by triggering AS of *TGFBR2* to regulate canonical TGF‐β signalling cascades differently. Our study provides a valuable transcriptome resource for myogenesis and reveals the complexity of AS and its regulation during myogenesis.

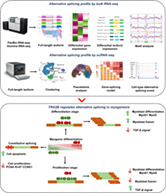

## 1 INTRODUCTION

Skeletal muscle is a crucial organ that enables animal movement, ingestion and metabolic processes, constituting over 30% of the overall mass of vertebrates.^1^ During embryonic myogenesis, muscle progenitors migrate to the limb bud terminal, where they undergo differentiation and generate muscle tissue under the regulation of various signalling pathways. Upon reaching the final destination, muscle progenitors undergo proliferation, differentiation into myoblasts and initial fusion to generate primary myotubes, characterised by a limited number of myocyte nuclei. The number of foetal myoblasts with enhanced proliferative capacity increases rapidly, utilising primary muscle fibres as a scaffold before amalgamating and differentiating into multinucleated muscle fibres.^2^ The development of skeletal muscle is regulated by a complex molecular network that includes transcription factors and signalling molecules. This network controls many aspects of myoblast biology, such as migration, proliferation, differentiation and fusion, as well as the differentiation of myogenic progenitor cells. The most important muscle‐specific regulators, *MYF5*, *MyoD1*, *MyoG* and *MRF4*, bind to the promoter E‐box motif to activate downstream target genes.^3^ Understanding the molecular mechanisms that regulate skeletal muscle development would be invaluable for researchers studying neuromuscular diseases, and could provide useful molecular markers for improving meat production in farm animals.^4^

In addition to myogenic transcriptional regulation, skeletal muscle growth and development are associated with extensive alternative splicing (AS) transitions.^5^ AS is critical for controlling gene expression and generating proteome diversity.^6^ AS regulates around 60% of avian genes^7^ and 90% of human genes.^8^ AS generates distinct transcripts by joining the original transcript and employing various exon combinations, thereby generating protein isoforms with distinct structures and ultimately regulating distinct cellular processes, such as cell proliferation, differentiation and apoptosis.^6^, ^9^ AS often occurs in a cell‐ or tissue‐specific manner. High‐throughput sequencing revealed that skeletal muscle and brain had the highest number of alternatively spliced transcripts.^10^ Several genes produce different protein isoforms by retaining unique pre‐mRNA sequences through AS.^11^ Abnormal regulation of AS can result in severe disease. Myotonic dystrophy types 1 and 2 typically exhibit a large number of anomalous AS events.^12^ Understanding the AS profiles during muscle development is essential for comprehending embryonic muscle fibre formation.

The regulation of AS is mainly controlled by *cis*‐regulatory elements located in pre‐mRNA and trans‐acting factors that bind to these elements.^13^ Among these trans‐acting factors, the SR (Serine/arginine‐rich splicing factor)^14^ and hnRNP (Heterogeneous nuclear ribonucleoprotein) families^15^ are the primary components. In addition, various RNA binding proteins (RBPs), including but not limited to *CELF*, *RBFOX1/2* and *MBNL* families, contribute to the control of AS. These proteins exhibit alternations in expression during distinct phases of tissue development, thereby leading to unique RNA splicing patterns.^16^ The splicing code of an RNA target is primarily influenced by the cell type, which determines the expression of specific RBPs and the distribution of AS transcripts across different cell types.^17^ To better understand the AS profile modulated by RBPs in diverse cellular contexts, it is crucial to use high‐throughput methodologies.

Sanger sequencing is a well‐established method considered the gold standard for gene expression level studies. It has been historically used for genome assembly annotation as well.^18^ However, short‐read sequencing has limitations in discovering identical exonic regions between different transcripts or the combination of complex splice sites, which can limit the identification of genetically variable spliced transcripts.^19^ In contrast, isoform long‐read RNA‐seq (Iso‐seq) enables the acquisition of full‐length transcript structures without assembly by forming uninterrupting RNA molecules. This technology provides advanced evidence to support AS studies and can significantly enhance the accuracy of existing gene model annotation.^20^

The use of single‐cell RNA (scRNA) sequencing has greatly expanded our understanding of continuous biological changes in gene expression and cellular heterogeneity.^21^ However, most scRNA‐seq analyses focus solely on gene expression arrays, which may overlook valuable information on transcriptional complexity.^22^ While full‐length scRNA‐seq methods like Smart‐seq2 offer complete isoform sequences, they are limited by high‐costs and low throughput. To address this issue, several AS detection methods have been developed for short‐read scRNA‐seq data sets using nanodroplet technologies, such as Sierra^23^ and MARVEL.^24^ Sierra identifies differential transcript usage (DTU) using an adapted differential exon usage testing method,^25^ while MARVEL employs a splice junction‐based approach for AS estimation. These AS detection approaches for short‐read scRNA‐seq data sets will enable the study of AS landscape and mechanism during myogenesis at cell‐type levels.

In this research, we generated an AS profile during embryonic myogenesis by utilising full‐length isoform sequencing on the PacBio platform, in combination with Illumina‐based short‐read sequencing and scRNA‐seq. Our analysis led to the discovery of differentially expressed transcripts produced by *TRA2B* during myoblast differentiation. Intriguingly, we also identified a novel transcript of *TRA2B* that plays an opposing role to the full‐length isoform during myogenic differentiation. These findings will contribute to a better understanding of AS during myogenesis.

## 2 RESULTS

### 2.1 The integration of Iso‐Seq and short‐read RNA sequencing provides a comprehensive view of the transcriptome during myogenesis

**FIGURE 1:**
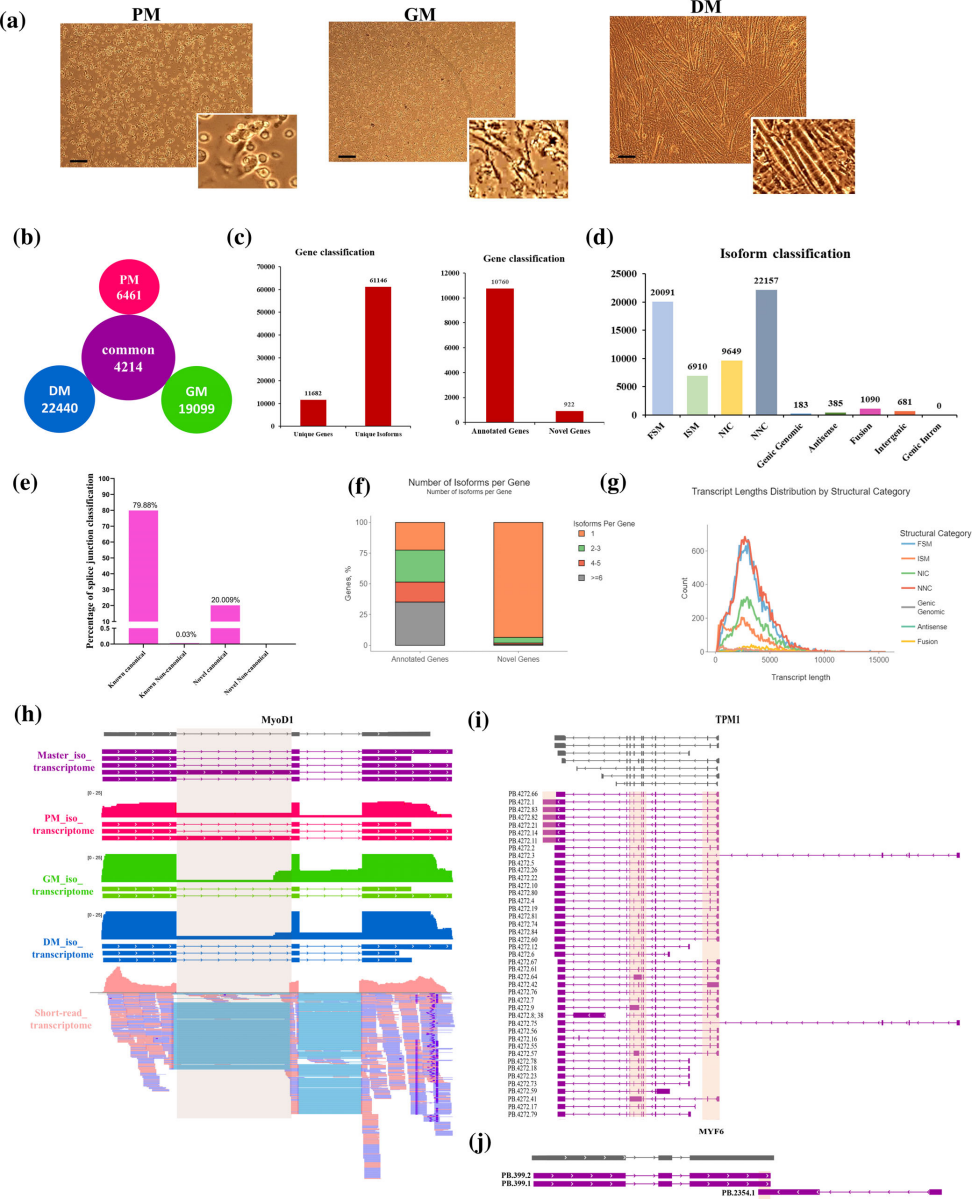
The integration of Iso‐Seq and short‐read RNA sequencing provides a comprehensive view of the transcriptome during myogenesis. (A) The PM, GM, and DM myoblasts were used for long‐read RNA‐seq. The morphology of three kinds of cells was determined by image analysis. The PM cells were oval and unstretched, while GM cells were extended with an expanded number. The GM cells reached indicated density, then began fusion together into a myotube with multiple nuclei. (B) The distribution of the number of common and specific full‐length isoforms in PM, GM and DM myoblasts. (C) The number of unique genes and isoforms resulted from transcript de‐redundancy analysis among the three stages of myoblasts. (D) In‐depth characterisation of isoforms using SQANTI3 based on isoform category. All isoforms were grouped into nine categories by SQANTI3. (E) Percentage of splice junction classification. (F) Number of variants for annotated and novel genes. (G) Transcript length distribution of seven structural categories. (H) Isoform maps of representative gene *MYOD1* detected by long‐read RNA‐seq and short‐read RNA‐seq. Orange rectangles indicate regulated splicing regions. Grey transcript retrieved from Ensembl GRCg6a. (I, J) Visualisation of full‐length isoforms mapped to master transcriptome constructed by PM, GM and DM long‐read transcriptome. Multiple splicing forms and transcript structures can be found in the same gene. DM, differentiated myoblast; FSM, full‐splice match; GM, growing myoblast; ISM, incomplete splice match; NIC, novel in catalogue; NNC, novel not in catalogue; PM, primary myoblast.

The chicken embryo is an excellent model system for studying skeletal muscle development. To investigate the complexity of the transcriptome during muscle fibre formation, we isolated the chicken primary myoblasts and cultured them in either growth medium for proliferation or differentiation medium for differentiation. To obtain a comprehensive overview of full‐length transcripts in different myogenic statuses of myoblasts, we performed PacBio long‐read RNA sequencing (Iso‐Seq) on primary muscle cells at PM, GM and DM stages (Figure 1A). In addition, we conducted RNA sequencing based on Illumina short‐reads to determine gene expression, given the adequate sequencing depth.^26^ In total, we generated 17,760,242 raw reads, which produced an average of 492,194 circular consensus sequences (CCSs) per phase (Table S1). We filtered and categorised these CCSs data as either full‐length non‐chimeric (FLNC) or non‐full‐length (nFL) sequences. We used LoRDEC to rectify Illumina‐based short reads with full‐length reads (Figure EV 1). We obtained a total of 461,494 consensus reads with an average length of 2964 bp after correcting errors (Table S1), which resulted in longer reads than the uncorrected refined consensus reads. The corrected isoforms were aligned to the chicken genome (Ensembl GRCg6a) using minimap2 (Figure 1B), and quality control and isoform annotations were conducted using SQANTI3 (https://github.com/ConesaLab/SQANTI3). To achieve a comprehensive transcriptomics profile of myogenesis, we combined the Iso‐Seq data sets of PM, GM and DM to create a master transcriptome (Figure EV 1; Dataset EV 2). After filtering with SQANTI and annotating isoforms, we recovered the master transcriptome comprising 61,146 isoforms that correspond to 11,682 unique genes (annotated genes: 10,760 [92%]; novel genes: 922 [8%]; Figure 1C). In contrast, the Ensembl transcriptome (GRCg6a) contained a total of 24,356 genes and 39,288 transcripts, indicating that our Iso‐Seq results revealed a greater diversity of AS isoforms within a single gene. Our analysis of the master transcriptome revealed a lower number of genes, but a greater number of isoforms compared to the Ensembl annotation. This suggests that a significant proportion of isoforms were novel and not previously catalogued. Around 33% of these unique isoforms were full‐splice matches (FSMs), while over 52% were either novel in or not in the catalogue (NIC and NNC), and did not match previously annotated Ensembl isoforms (Figure 1D). Splice junction identification showed that 80% of isoforms used known canonical splice sites, while the remaining 20% used novel canonical splice sites (Figure 1E). The master transcriptome also showed that over 70% of genes had two or more isoforms (Figure 1F), which was higher than the Ensembl transcriptome. The transcript length of the structural category mostly ranged between 1 and 2 kb, consistent with the length of corrected isoforms (Figure 1G). We also analysed the Iso‐Seq of PM, GM and DM using SQANTI3, yielding a total of 23,418, 24,664, and 28,009 isoforms, respectively (Figures EV 2–4). Most of the isoforms identified belonged to the FSM, ISM, NIC and NNC categories (Figures EV 2–4A,B). Regardless of the period, transcript length, number, and coding capacity were consistent with the master transcriptome (Figures EV 2–4C–F and EV 5A). The majority of PM, GM and DM isoforms differed from the annotated transcription start site and transcription termination site by an average of 100 bp (Figure EV 2–4I,J), indicating novel isoforms with distinct internal splice junctions. This finding was supported by *MyoD1* transcripts obtained from Iso‐Seq and short‐read RNA‐seq, which revealed an intron‐retaining isoform (Figure 1H and Figure EV 5B). While short‐read RNA‐seq data aligned to the region of intron 1, it could not identify the specific isoform the read belonged to, highlighting the advantages of Iso‐Seq. *TPM1* and *PDLIM7* are two genes with important roles in muscle development and muscular diseases. We detected various isoforms in *TPM1* and *PDLIM7*, which included transcript fusion, alternative first exon, alternative last exon, intron retention and exon exclusion (Figure 1I and Figures EV 5D and EV 6A–C). Additionally, we identified known exclusive exons in *MEF2C* (Figure EV 5C), and novel antisense transcripts near known genes, such as *MYF6* (Figure 1J). During myoblast proliferation and differentiation, we also observed global alternative polyadenylation (APA). Interestingly, the distribution of gene numbers for APA remained constant in each period (Figure EV 6D–F). Overall, our use of Iso‐Seq and short‐read RNA‐seq provided an overview of the transcriptome during myogenesis (Figure EV 6G).

### 2.2 Myogenic differentiation‐dependent AS dynamics differ significantly from gene expression profiles

**FIGURE 2:**
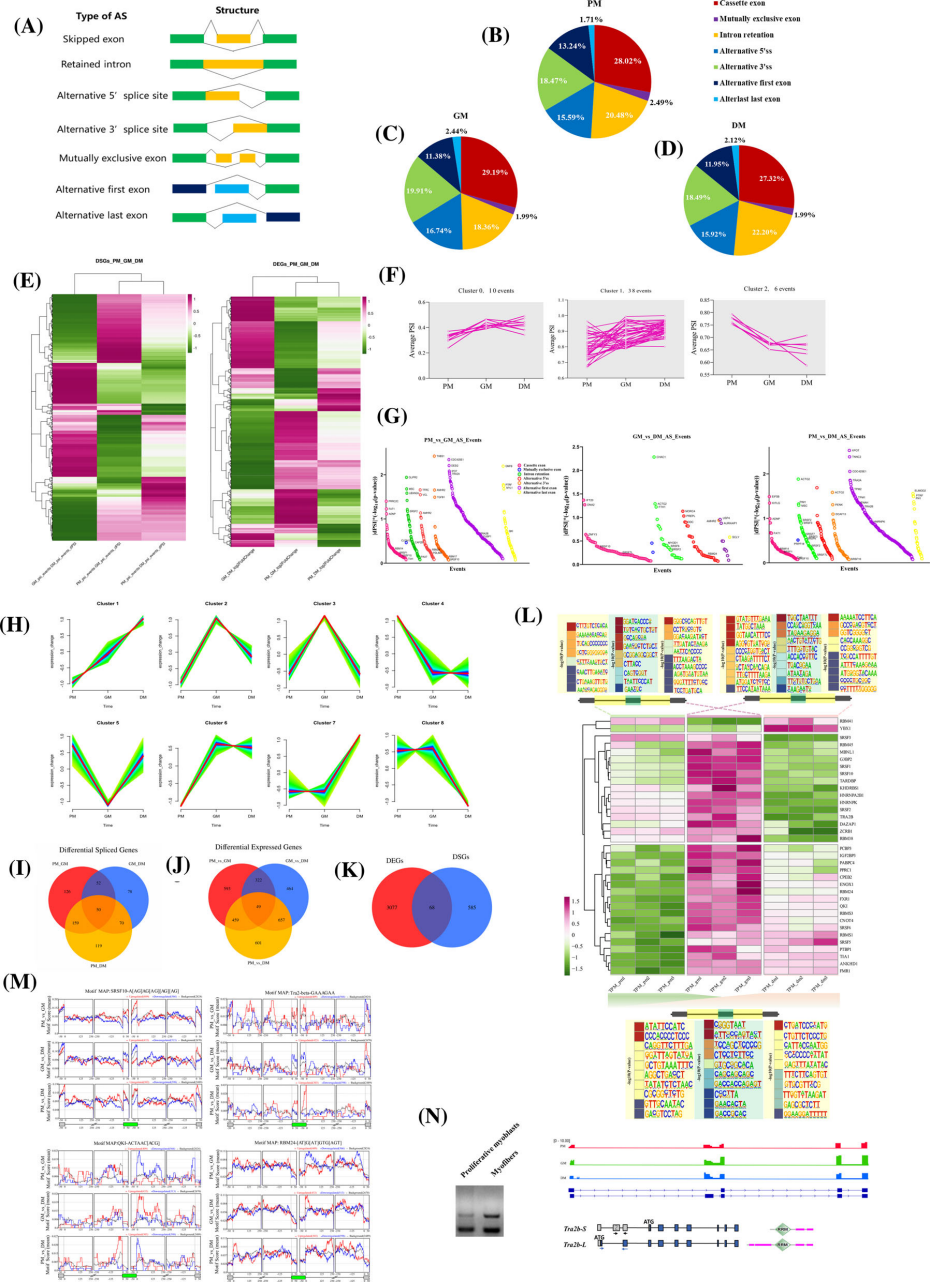
Myogenic differentiation‐dependent alternative splicing dynamics differ significantly from gene expression profiles. (A) Classification of AS events using SUPPA2. The number of AS events detected by SUPPA2 for PM myoblasts (B), GM myoblasts (C) and DM myoblasts (D). (E) Heatmaps showing the differentially expressed genes (DEGs) and differentially spliced genes (DSGs) between PM, GM, and DM. The DEGs were screened as |log_2_FoldChange| > 2 and *p*adj < 0.05. The DSGs were genes that differentially spliced with PSI > 0.01 and *p* value <0.05. (F) AS events with similar PSI changes between the three stages were calculated using density‐based clustering. (G) The DSGs are between two stages. (H) Temporal dynamics cluster analysis based on TPM of expressed genes during PM, GM and DM myoblasts. (I–K) Venn diagram of DSGs (I) and DEGs (J) between any two stages, and a Venn diagram showing DEGs and DSGs between three stages (K). (L) Motif enrichment in intronic regions flanking regulated and AS exons between the two stages. Heatmaps showing the relative expression change of RNA‐binding proteins during myoblast proliferation and differentiation were clustered into three levels of expression patterns. (M) RNA‐binding proteins‐CLIP map showing regional binding of indicated RNA‐binding proteins with specific motifs in the 250 bp flanking introns of skipped and included exon events. The number of splicing events between two consecutive stages is indicated. The background measurement shown as a black line was calculated using all non‐significantly spliced exons. The red and blue lines represented the increased inclusion and decreased inclusion in AS events, respectively. (N) RT‐PCR splice products for AS events of *TRA2B* comparing proliferative myoblasts to myotubes. The full‐length *TRA2B* protein (*TRA2B‐L*) is encoded at the beginning of the first exon, while the truncated isoform is encoded from the fourth exon (*TRA2B‐S*). AS, alternative splicing; DM, differentiated myoblast; GM, growing myoblast; PM, primary myoblast; PSI, percent spliced in; RT‐qPCR, reverse transcription‐quantitative real‐time PCR.

AS acts as an essential regulator for gene expression and protein diversity. Skipped exon (SE), retained intron (RI), alternative 5′ splice site (A5SS), alternative 3′ splice site (A3SS), mutually exclusive exon (MX), alternative first exon (AF) and alternative final exon (AL) are the seven fundamental kinds of AS formation (Figure 2A). Using Iso‐Seq, we identified 38,022 AS events in the master transcriptome. Across all three time periods, SE had the largest percentage of AS events, followed by RI, A5SS and A3SS (Figure 2B–D). Ten SE events were chosen at random to test the authenticity of skipped exons using RT‐PCR, proving the precision of Iso‐Seq in identifying AS events (Figure EV 7). To delineate stage‐associated AS events and transcriptional dynamics during myogenesis, we conducted a screening of differentially spliced genes (DSGs) and differentially expressed genes (DEGs) between three stages of myoblast development: primary myoblast (PM), growing myoblast (GM) and differentiated myoblast (DM). The number of significantly spliced events was found to be higher in PM compared to GM or DM (Figure 2E; Dataset EV 3). We identified 1050 spliced events across all stages, and 3145 DEGs (*p*adj < 0.05 and |log2FC| > 2) enriched in cell development, cell adhesion, muscle cell differentiation and ECM–receptor interaction (Figure 2E and Figure EV 8A–C). The DSGs between PM, GM and DM were mainly enriched in the regulation of RNA splicing, cytoskeleton regulation, regulation of cell cycle and spliceosome (Figure EV 8D–F). We conducted density‐based clustering of percent spliced in (PSI) change between any two phases, which showed that most AS events were initially dynamically up‐ or downregulated, while the splicing level of cassette exons progressively converged (Figure 2F). The genes that show significant variability across AS events may not be constant over time (Figure 2G). To investigate changes in gene expression, we grouped all genes into eight clusters based on the expression change (Figure 2H). The clusters with the highest gene counts were clusters 2, 3 and 7. Clusters 2 and 3, which consist of 3168 and 4265 genes, respectively, showed increased expression levels in GM but were downregulated in DM (Figure 2H). When comparing DEGs or DSGs between any two stages, we found that only a small number of genes were present in all three comparisons (Figure 2I–K). This suggests that transcriptional and post‐transcriptional alterations are dependent on myoblast development. Interestingly, most DEGs and DSGs during PM, GM and DM did not overlap (Figure 2K), indicating that differential gene expression and AS are both dynamic during myoblast development in a stratum‐specific manner. Furthermore, most splicing events occur within open‐reading frames (ORFs) rather than a change in gene expression.

To gain deeper insights into the mechanisms of splicing transition during myoblast proliferation and differentiation, we investigated the expression dynamics of RNA‐binding proteins (RBPs) that are conserved across vertebrates during the transition of PM, GM and DM. Results showed that most RBPs were differentially expressed during myoblast proliferation and differentiation (Figure 2L,M). To identify the *cis*‐acting regulatory elements for cassette exons of DSGs, we extracted a 200‐bp flanking sequence around the alternative spliced exon within the intron and SE sequence, which revealed several enriched motifs within the upstream and downstream regions of the intron and skipped exon (Figure 2L). Using short‐read RNA‐seq to detect the binding sites of RBPs, we found the effect of the RBPs on AS largely depends on their preference relative to the alternatively spliced region (Figure 2M). Interestingly, we observed a significant enrichment of the *TRA2B* binding sequence (5′‐GAAGAA‐3′) in the SE, which was supported by both Iso‐Seq and short‐read RNA‐seq (Figure 2L,M). Besides, our findings suggest that *TRA2B* may regulate splicing programs through interaction with other RBPs (Figure EV 8G). Interestingly, we also found that an increase in the expression of a skipped exon during myoblast differentiation, resulting in a truncated isoform lacking the first RS domain, which we named *TRA2B‐S* (Figure 2N).

### 2.3 scRNA‐seq revealed cellular heterogeneity and signatures of myogenesis

**FIGURE 3:**
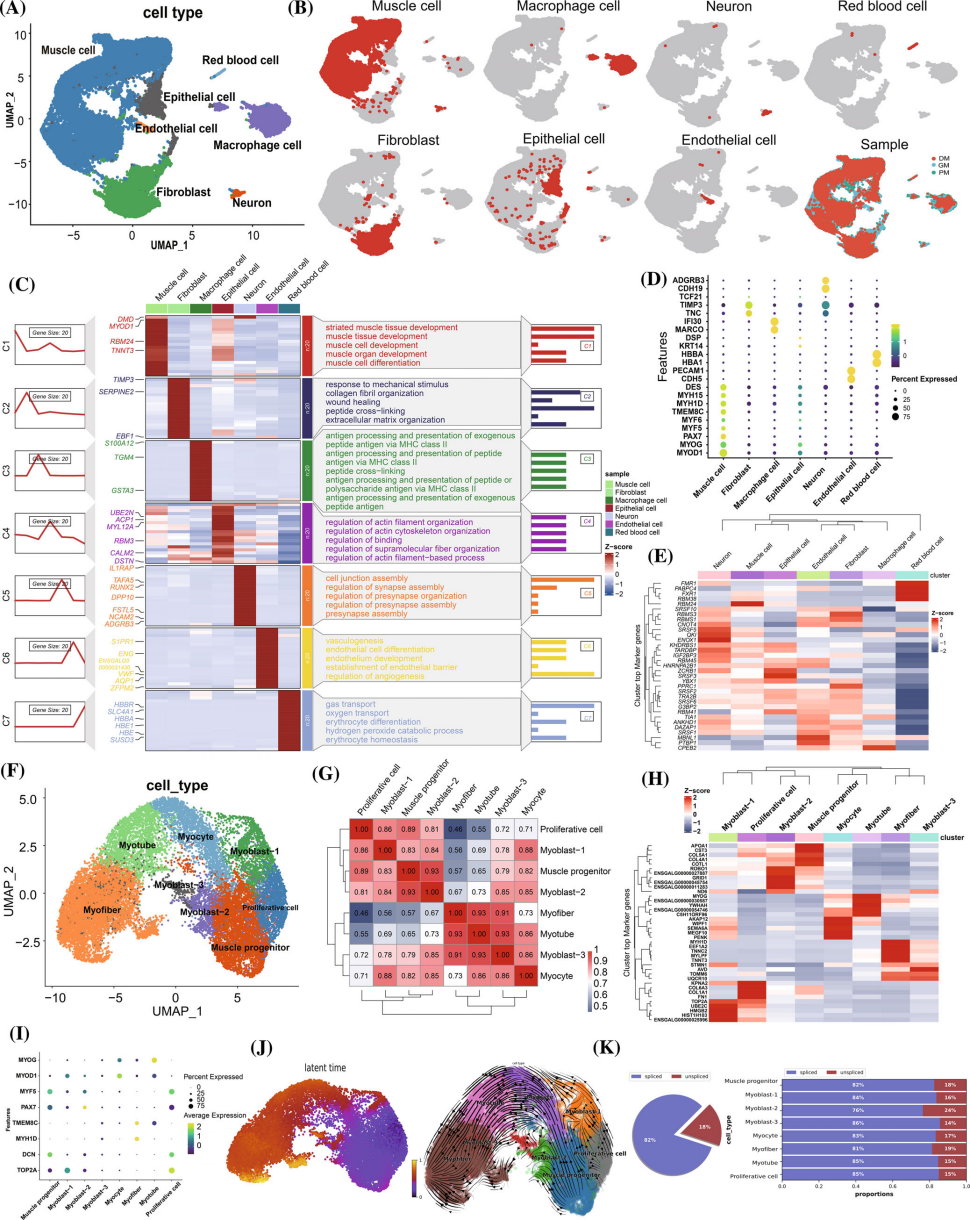
scRNA‐seq revealed cellular heterogeneity and signatures of myogenesis. (A) An unsupervised UMAP plot embedding 32,778 cells from PM, GM and DM stages that clustered and annotated by cell type. Each point represents a single cell. (b) UMAP plot showing the distribution of each cell type. The last UMAP plot represents the cells that are integrated by stages of PM, GM and DM. (C) Fuzzy c‐means clustering of gene expression, differentially expressed genes and functional enrichment for different cell types. (D) Dot plot showing the expression of representative marker genes for each cell type. (E) Heatmap showing the expression of RNA binding proteins across cell types. (F) UMAP plot showing the subpopulations of 20,632 muscle cells. Cells are coloured according to annotated cell types. (G) Muscle cell types were grouped into functional clusters by Spearman correlation. The colour intensity represents the correlation value. (H) Top six DEGs of each cell type of muscle cell visualised by heatmap. (I) Dot plot showing the expression of representative genes across cell subpopulations of muscle cells. (J) Latent time and RNA‐velocity analysis inferred the differentiation trajectories. (K) The percentage of spliced/unspliced counts. Genes that started to be transcribed were reflected by the proportion of unspliced counts at cell fate commitment of specific cell types. DEG, differentially expressed gene; DM, differentiated myoblast; GM, growing myoblast; PM, primary myoblast; UMAP, Uniform Manifold Approximation and Projection.

To gain insight into the degree of heterogeneity of muscle cells and the progressive profiling of the myogenic differentiation process, we collected samples from the PM, GM and DM stages. For each sample, cells were enzymatically digested and processed into a single‐cell suspension, which was then fed into the scRNA‐seq 10× Genomics Chromium. After quality control, 32,778 cells were used for downstream analysis (14,387 at PM, 7992 at GM and 10,399 at DM), with a median of 6766 unique molecular identifiers (average: 6794) and 2296 genes per cell (average: 2116) (Figure EV 9A). To classify cell populations in PM, GM and DM samples, we performed unsupervised clustering using the Uniform Manifold Approximation and Projection (UMAP) and t‐Distributed Stochastic Neighbour Embedding (t‐SNE) method. This approach enabled us to identify major cell types based on the expression of canonical marker genes. The identified cell types include muscle cell (20,632), epithelial cell (1894), endothelial cell (149), fibroblast (6267), macrophage cell (3422), neuron (272) and red blood cell (142) (Figure 3A and Figure EV 9C). Based on UMAP and t‐SNE results, all cell types were detected in all three sample phases, indicating no pronounced batch effect (Figure 3B, last panel and Figure EV 9B,D–F).

Transcriptome changes for each cell type were also revealed. A heatmap comparing DEGs between cell types showed that transcriptomic differences are closely associated with cell‐type clusters (Figure EV 10A; Dataset EV 4). Specifically, 649 genes were found to be differentially expressed between muscle cells and other cell types (Dataset EV 4). Gene ontology (GO) analysis suggested that the genes highly expressed in muscle cells, including striated muscle contractile element gene (*DMD*, *TNNT3*, *MYH1D*, *DES* and *MYH15*), canonical MRFs (*MYOD1*, *MYOG* and *MYF5*) and regulatory genes (*PAX7* and *TMEM8C*), play key roles in regulating and participating in muscle tissue development and muscle cell differentiation (Figure 3C,D and Figure EV 10B). Differential genes and enrichment pathways of other non‐muscle cell subpopulations were also identified (Figure 3C,D and Figure EV 10C–H). GSVE analysis showed that the gene sets of muscle cells were significantly enriched in myogenesis and MYC pathway (Figure EV 10I). This result further supports the reliability of our annotated muscle cell types. Additionally, we observed that cell types with similar phenotypes had higher correlations in their gene expression profiles. Other cell types are more distantly related than muscle cells, epithelial cells, and fibroblasts (Figure EV 10J). The results of the correlation analysis of gene expression patterns showed that muscle cells, fibroblasts and epithelial cells had more strongly correlated gene expression patterns than other subgroups, indicating that these three cell types share more similarities in their gene expression profiles (Figure EV 10J). Specifically, RBPs known to be involved in muscle‐related processes, such as RBM24, RBM38, SRSF10 and QKI, were highly expressed in muscle cell clusters compared to other cell types (Figure 3E and Figure EV 10K), suggesting a potential role for these RBPs in muscle cells.

### 2.4 Cellular state transition and gene regulation during myogenic differentiation

Through unsupervised clustering and cell‐type annotation analysis, we subclustered 20,632 muscle cells into eight cell types, including proliferative cell (*TOP2A+*/*MYF5+*/*DCN*+), muscle progenitor (*PAX7*+/*MYF5*+/*DCN*+), myoblast‐1 (*TOP2A*+/*MYOD1*+), myoblast‐2 (*PAX7+*/*MYF5+*/*MYOD1+*), myoblast‐3 (relative low expression of *MYOD1*/*PAX7*/*MYF5* and highly expressed *DES*), myocyte (*MYOD1*+/*MYOG*+), myotube (*MYOG*+/*MYOD*+/*TMEM8C*+) and myofibre (*MYH1D*+/*TMEM8C*+) (Figure 3F,I). We assessed the marker gene activity in each cell subpopulation, and the results indicate that the identification of cell subpopulations is reliable (Figure EV 11). Consistent with the UMAP dimensionality reduction and unsupervised clustering, the adjacent clusters have a higher correlation than distant clusters, indicating that cells within each cluster share similar gene expression profiles and potential evolutionary relationships (Figure 3G). Analysis of differentially highly expressed genes in the subpopulation revealed a distinct pattern of gene expression (Figure 3H; Dataset EV 5). All posterior cells originated from muscle progenitor cells were highly expressed genes involved in extracellular matrix organisation and cell‐substrate adhesion, such as *DCN*, *COL4A1*, *COL5A1* and *COL3A1* (Figure 3H and Figure EV 12A–C). Other than muscle progenitors, a proliferative progenitor cell population was identified, which displayed elevated expression of *TOP2A*, *CCNB1*, *PCNA*, *DCN*, *MYF5* and *PAX7*, indicating their ability to maintain pluripotency (Figure 3I and Figure EV 12A). Compared to GO terms enriched in muscle progenitor cells, the highly expressed genes in proliferative cells were primarily associated with nuclear division, mitosis and microtubule cytoskeleton organisation (Figure EV 12C). Myoblasts derived from muscle progenitor and proliferative cell clusters were identified by unsupervised clustering and expression of canonical markers. There were 756, 651 and 53 genes significantly enriched in myoblast‐1, myoblast‐2 and myoblast‐3, respectively (Dataset EV 5). Myoblast‐1 maintained its proliferative and migratory capacity, as indicated by highly expressed marker genes *PCNA* and *HMGB1*, as well as enriched GO terms for mitosis regulation and DNA metabolic process (Figure EV 12A,C). Calcium ion regulation, PKB signalling and cytoplasmic translation were significantly represented in myoblast‐2/3, with a distinction observed between myoblast‐2 and myoblast‐3. Myoblast‐2 expressed higher levels of *PAX7*, *MYF5* and *MYF6* and was more closely related to muscle progenitor cells, while myoblast‐3 expressed higher levels of structural proteins such as *TNNT2*, *TNNC1*, *TNNC2* and *DES* and was more closely related to myotube (Figure 3F,G; Dataset EV 5). Myocytes exhibited high *MYOD1* and low *MYOG* expression, indicating that the capacity for cell fusion progressively increased (Figure 3I and Figure EV 11B). The myocyte cluster was heavily represented by GO terms associated with myoblast differentiation and skeletal muscle cell differentiation (Figure EV 12C). Notably, *MYOG* expression was found to be greatest in myotube clusters. In the meantime, the majority of cells in myotube clusters expressed *TMEM8C*, a fusion marker gene, indicating that cell fusion had begun (Figure 3I and Figure EV 11B). Genes highly expressed in myotubes were enriched in positive regulation of myoblast and myotube differentiation, striated muscle cell differentiation and protein‐containing complex assembly (Figure EV 12C). In the myofibre cluster, the expression of contractile proteins and myosin filaments was higher (Figure 3H,I and Figure EV 11A,B). GO analysis revealed significant enrichment of muscle contraction, myofibril assembly, and actin‐myosin filament sliding in the myofibre cluster (Figure EV 11C).

In addition, we conducted a GSVA analysis using the gene expression profile of each subcluster of muscle cells. This analysis revealed the enrichment of several canonical signalling pathways, such as Wnt‐β‐Catenin, TGF‐β, MYC, myogenesis and cell cycle regulation, indicating their potential role in specific cell types (Figure EV 12D). Besides, we investigated the expression of RBPs across cell types. Our findings showed that the expression of RBPs, including *YBX1*, *SRSF1*, *TIA1*, *HNRNPA2B1* and *ANKHD1*, was comparable between cell types. Simultaneously, RBPs such as *TRA2B*, *RBM24*, *PTBP1*, *SRSF10*, *FXR1* and *RBM38* displayed a dynamic pattern between cell types, suggesting their activation and function in specialised cell types (Figure EV 12E,F). Furthermore, the progression of latent time reconstructed the differentiation of myogenic cells in stages, with muscle progenitor and proliferative cell acting as early points of differentiation and myofibre being the final product (Figure 3J). Notably, the ratio of spliced and unspliced mRNA counts was lower in myoblast‐2 and myofibre than in other cell types, suggesting that the transcription of genes is more active in these cells (Figure 3K). Using an unsupervised algorithm Monocle3, we reconstructed the pseudotime order of muscle cell subcluster. This order was found to be consistent with RNA velocity results (Figure EV 12G). We observed that as cells differentiated along the lineage trajectory, the expression of genes involved in muscle contraction increased, while the proliferation marker *TOP2A* progressively decreased (Figure EV 12H). Overall, our scRNA‐seq analysis provided a comprehensive transcriptomic profiling of the cellular transition that occurs during myogenesis.

### 2.5 Cell‐type‐specific AS shaping cellular identity and transcriptome diversity

**FIGURE 4:**
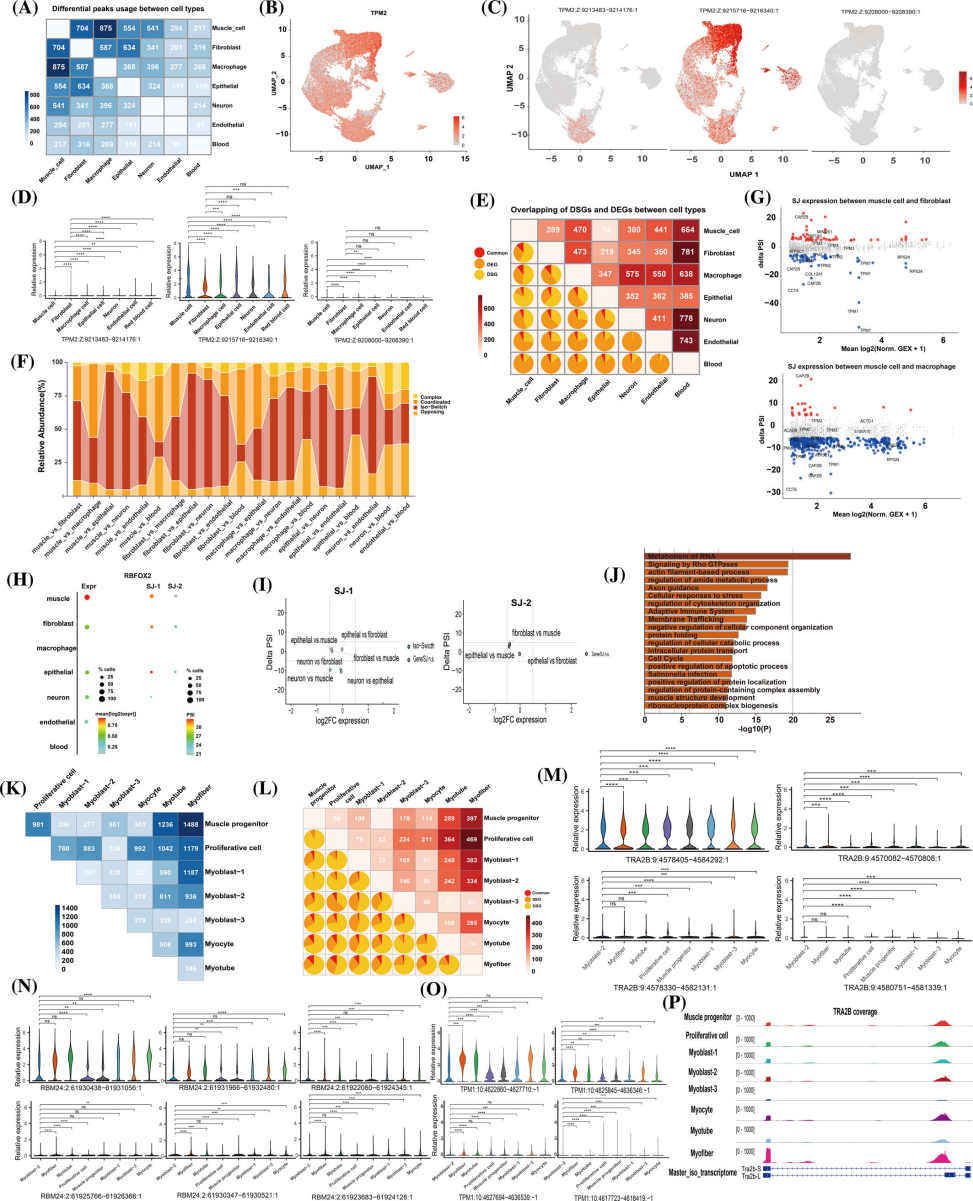
Cell‐type‐specific alternative splicing events contribute to cell identity and augment transcriptome diversity. (A) Number of differential usage peaks between cell types. (B) Expression of TPM2 gene projected onto the UMAP plot. (C) Feature plot showing relative peak expression for differential usage peaks of TPM2. (D) Violin plot showing the relative expression of differential usage Peaks across cell types. (E) Overlapping of differentially expressed genes (DEGs) and differentially spliced genes (DSGs) that possess differential peaks usage between cell types. The number of differentially expressed genes was shown in the top right portion. The percentage of DEGs, DSGs and common genes was shown in lower left portion. (F) The percentage of gene‐splicing dynamics between cell types. Gene‐splicing dynamics that integrated gene expression and splice junction usage were classified into coordinated, opposing, isoform‐switching (iso‐switch) and complex categories. (G) Volcanic map showing PSI difference of splice junction usage between muscle cell and fibroblast (left panel) or muscle cell and macrophage (right panel). Red points represented the expression of splice junctions increased in muscle cells (delta PSI > 5 and *p* value <0.05), while the blue points indicated the splice junctions decreased in muscle cells (delta PSI < −5 and *p* value <0.05). (H) Expression of *RBFOX2* gene and splice junctions across cell types. (I) Alteration of splice junction usage compared to gene expression between cell types. (J) Muscle‐relative genes with differential spliced transcripts were used to functional enrichment analysis. (K) Number of differential usage peaks between muscle cell subpopulations. (L) Overlapping of DEGs and DSGs that possess differential peaks usage between subtypes of muscle cells. (M–O) Violin plot showing the relative expression of differential usage Peaks across cell types. (P) coverage plot of specific AS events of TRA2B across cell types. The differences between groups were evaluated using the Wilcoxon test for statistical analysis. **P < 0.01, ***P < 0.001, ****P < 0.0001. PSI, percent spliced in; UMAP, Uniform Manifold Approximation and Projection.

After demonstrating the cellular heterogeneity during myogenesis via gene expression clustering, we aimed to investigate AS differences between cell types through transcript expression using established algorithms. Sierra was utilised to identify DTU between cell types by calculating the relative usage of peaks located within distinct gene regions.^23^ To estimate PSI, we employ a splice junction‐based approach from splice junction (SJ) reads to evaluate the AS of the specified cell types.^24^ Significant DTU between different lineages was detected by pairwise comparisons using peaks falling on 5′UTRs, 3′UTRs, exons or introns (adjusted *p* < 0.05, logFC > 0.25). A total of 7453 DU peaks corresponding to 4872 DTU genes were identified in all pairwise comparisons. Notably, the muscle cell and macrophage cluster had the greatest number of DTU peaks (875), corresponding to 602 DTU genes with at least one DTU peak (Figure 4A and Figure EV 13). We also compared muscle cell and fibroblast samples and found a total of 704 distinct DTU peaks, representing 462 DTU genes (Figure 4A and Figure EV 13). Interestingly, we observed that *TPM2*, a member of the actin filament binding proteins, was ubiquitously expressed among muscle cells, fibroblasts, and epithelial cells. However, two DTU peaks of *TPM2* were preferentially expressed in the muscle cells and fibroblasts, respectively (Figure 4B–D). Specifically, the peak (TPM2:Z:9213483–9214176:1) was significantly higher in the fibroblast subcluster compared to other cell types, while the peak (TPM2:Z:9215716–9216340:1) was higher in muscle cell compared to other subclusters (Figure 4B–D). These results demonstrated that Sierra detected DTU of *TPM2* gene in fibroblast and muscle cell populations, which corresponds to the alternative transcript expression events.

We compared the relationship between DTU genes and DEGs within the same comparisons. Across the two groups of cell clusters, regardless of comparison, the proportion of genes that belong to both DTU genes and DEGs genes is consistently the lowest, which aligns with the findings from long‐read RNA‐seq (Figure 4E). Using gene‐splicing dynamics (Section 4), we determined the relationship between AS and gene expression. Among all four gene‐splicing dynamics, isoform‐switch dynamics, which represent genes that are differentially spliced but not differentially expressed, dominated approximately 57% of all pairwise comparisons (Figure 4F). To explore the splicing quantification method based on the spliced junction in depth, we compared the splicing differences between muscle cell subpopulations and other subpopulations. By differentially splice junction analysis, 111 (muscle cell vs. fibroblast), 499 (muscle cell vs. macrophage), 228 (muscle cell vs. epithelial cell), 863 (muscle cell vs. neuron), 763 (muscle cell vs. endothelial cell) and 179 (muscle cell vs. blood) differentially splicing events were identified, corresponding to 77, 330, 160, 452, 406 and 123 DSGs, respectively (Figure 4G and Figure EV 15C–F; Dataset EV 6). Notably, genes related to muscle contraction and RNA splicing showed differentially splicing across the comparison between muscle cells and other cell types (Figure EV 15A,B). It was reported that *RBFOX2* regulates the expression of the muscle‐specific MEF2D‐V4 transcript, which promotes the differentiation of myoblasts.^27^ *RBFOX2* was highly expressed in muscle cells. Splice junction‐1 (SJ‐1; chr1:51988309:51989385) showed a decrease in neurons compared to the muscle cells, while splice junction‐2 (SJ‐2; chr1:51987318:51988235) increased in fibroblast and epithelial cell (Figure 4H). The SJ‐1 usage of *RBFOX2* was associated with isoform‐switch in multiple cell comparisons, including between muscle cell and neuron, neuron and fibroblast, and neuron and epithelial cell (Figure 4I). There is minimal variation in the expression of SJ‐2 across all cell populations, suggesting a low degree of diversity. The genes that exhibited differential splicing patterns between muscle cells and other cell types were found to be significantly enriched in various biological processes, such as RNA metabolism, actin filament‐based processes, regulation of the cell cycle, apoptosis, and development of muscle structure. (Figure 4J). The splicing differences between different cell subgroups contribute to the heterogeneity of muscle tissue, and further understanding of AS events helps to decipher the mechanisms of muscle growth and function.

Subsequently, the splicing profile was evaluated across subclusters of muscle cells. In pairwise comparison of two cell types, a total of 17,952 differential usage peaks and 11,699 DTU genes were identified (Figure 4K and Figure EV 14). The most significant number of DTU genes and DTU peaks were screened out when comparing muscle progenitor and myofibre, suggesting an increased proportion of genes relative to muscle contraction and actin‐myosin assembly in myofibre (Dataset EV 7). The MAST testing revealed differential gene expression between subsets of muscle cells. It is noteworthy that a significant number of DEGs were found between cells that underwent pre‐differentiation and those that underwent post‐differentiation. This quantity was observed to be higher than the number of DEGs identified between two consecutive subpopulations of cells during the differentiation process (Figure 4L). The relationship between DEGs and DSGs in the same comparison of two cell types was explored. The common genes that were involved in both DEGs and DSGs only occupied up to 10% of the three categories (Figure 4L). The genes involved in RNA metabolism and the contractile system of striated muscle exhibited differentially spliced across subclusters. The DTU peak (9:4570082–4570808) of the *TRA2B* gene was found to be expressed preferentially in myofibre and myoblast‐2, and the peak (9:4578330–4582131) was expressed higher in myofibre compared to other cell types (Figure 4M). A muscle‐enriched RBP, *RBM24*, showed differential splicing across subclusters (Figure 4N). In DEXseq testing, actin‐binding proteins *TPM1*, *TPM2* and *TPM3*, which are related to contractile striated muscle and cytoskeleton, were significantly represented. *TPM1*, *TPM2* and *TPM3* were expressed across muscle progenitor to myofibre. For TPM1, specific peaks (TPM1:10:4622860–4627710 and TPM1:10:4617723–4618419) were distinguishable between myofibre and proliferative cell or muscle progenitor, while the other peaks were uniformly expressed across subclusters (Figure 4O and Figure EV 16A). Two DTU peaks of *TPM2* were highly expressed in all muscle cell types. One of the DTU peaks (TPM2:Z:9215715–9216339) was enriched in myofibre, whereas the other was expressed highly in all subpopulations (Figure EV 16B). Three DTU peaks of *TPM3* were also identified, two of which were expressed predominantly in myotube and myofibre, while the remaining one was mainly expressed in myoblasts, muscle progenitor, and proliferative cell (Figure EV 16C). *CAPZB* is involved in the regulation of cytoskeleton and sarcomere assembly, and its dysfunction can lead to disrupted myogenic differentiation and embryonic lethality.^28^ A differential usage transcript of *CAPZB* (21:4688766–4688994) showed an upregulation in myotube and myofibre, whereas another peak (21:4686598–4698829) was expressed extensively in various muscle subclusters (Figure EV 16D). Similar transcript expression patterns (1:105352154–105354258 and 1:105353034–105353238) were observed in the *CCT‐8* locus, which is required for actin and tubulin expression (Figure EV 16E).^29^ Furthermore, we compared the RNA‐binding proteins (RBPs) with splice junction expression changes during myogenic differentiation in the formation of multinucleated myofibres by muscle progenitor cells and found that *TRA2B*, *HNRNPA2B1* and *YBX1* are RBPs with variable splicing events during myogenic differentiation (Figure EV 17). Because a skipped exon was found in *TRA2B*, we visualised the relative peak expression in the local transcript. An enriched peak covering the alternative exon was observed and increased in myocyte and myofibre, while lower expression was observed in muscle progenitor and myoblast clusters (Figure 4P).

We next sought to assess the splicing difference between muscle cell subclusters by differential splice junction analysis. Among 28 pairwise comparisons, 6925 genes were defined as DSGs, comprising 11,019 different splice junctions (Dataset EV 8). Notably, genes that regulate cytoskeleton organisation and RNA splicing, such as *NEXN*, *NACA*, *TNNT1*, *TPM1*, *TPM2*, *TPM3*, *FXR1*, *HNRNPDL* and *SRSF7*, were differentially spliced between cell types (Figure EV 18; Dataset EV 8). To investigate the regulation between gene expression and AS modifications, the gene‐splicing dynamics between cell types were determined. Similar to previous findings, isoform‐switch was the dominant mechanism driving the observed relationships between muscle cell types. These results suggest that AS plays a critical role in myogenic differentiation and provides an additional regulatory mechanism beyond differential gene expression (Figure EV 19A). Next, we investigated the splice junction usage and gene expression of DSGs during muscle differentiation. Our findings revealed that *RBM24* was highly expressed in myocyte and myotube, while splice junction‐1 usage increased from myocyte and myotube to myofibre (Figure EV 19B). The splice junction (SJ‐1, SJ‐2, SJ‐3 and SJ‐4) usage of *TPM1* increased along myogenic differentiation, which was coordinated with *TPM1* expression (Figure EV 19C). The *TPM2* gene exhibited elevated expression levels in both the proliferative cell and muscle progenitor, and SJ‐1 and SJ‐2 of *TPM2* were notably abundant in the proliferative cell, muscle progenitor, myoblast‐1, and myocyte (Figure EV 19D). In addition, we discovered that SJ‐1 of *TPM3* was highly used in myofibre, while the total gene expression of *TPM3* was elevated in proliferative cells (Figure EV 19E). *NACA* is necessary for myofibril structure,^30^ and we found that the SJ‐1 of *NACA* was highly expressed in muscle progenitor, myoblast‐1, myoblast‐2 and myoblast‐3 clusters, which was inconsistent with its high gene expression in myocyte and myotube (Figure EV 19F). For RBPs involved in the regulation of AS, an isoform‐switch was identified in *SRSF7* and *FXR1*(Figure EV 19G,H,J). We also identified the splicing events in *CAPZB*, which was highly expressed in proliferative, myoblast‐1, myotube and myofibre cells. SJ‐1 usage of *CAPZB* increased in myoblast‐3, myotube and myofibre, while SJ‐2 usage remained consistent across all cell types (Figure EV 19I). Our investigation of the gene‐splicing dynamics of *TRA2B* revealed an isoform‐switch during myogenic differentiation (Figure EV 19KE). In summary, our analysis of DTU peaks and splicing junction usage strategy from scRNA‐seq data uncovered differences in gene expression and splicing dynamics between muscle subclusters.

### 2.6 Two isoforms of *TRA2B* are necessary for myoblast proliferation and play different roles in myoblast differentiation and myotube formation

**FIGURE 5:**
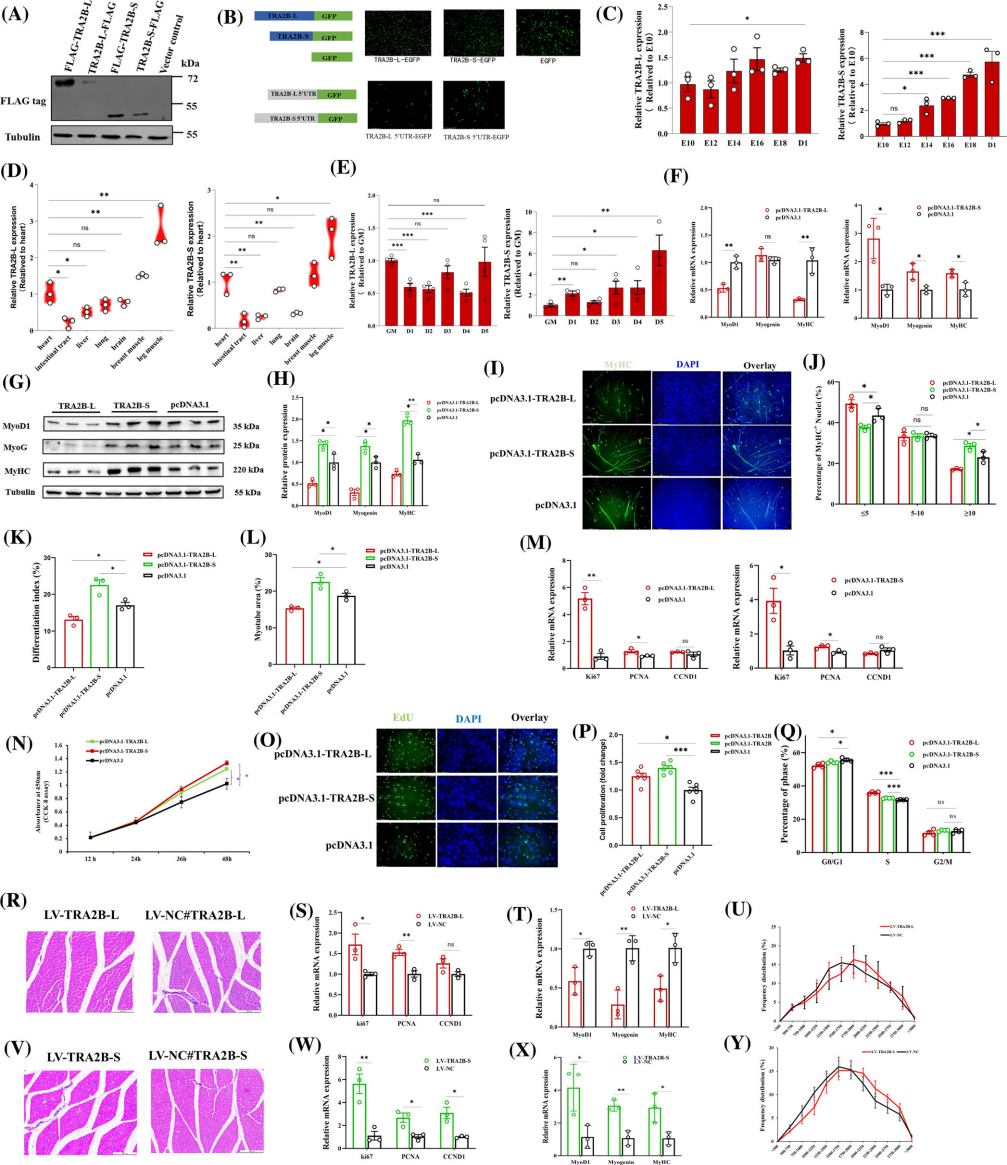
Two isoforms of *TRA2B* are necessary for myoblast proliferation and play different roles in myoblast differentiation and myotube formation. (A) The protein‐coding potential of two isoforms of *TRA2B* was determined by western blot. (B) The protein‐coding potential of two isoforms of *TRA2B* was determined by fluorescence. Upper: expression vector containing the sequence of TRA2B isoforms was transfected into myoblast. Lower: effect of 5′UTR of two AS isoforms on coding potential. (C) Expression changes of *TRA2B‐L* and *TRA2B‐S* during embryonic development (*n* = 3). (D) The expression of *TRA2B‐L* and *TRA2B‐S* in different tissues (*n* = 3). (E) The expression changes of *TRA2B‐L* and *TRA2B‐S* during myoblast differentiation (*n* = 4). (F) mRNA abundance of *MyoD1*, *Myogenin* and *MyHC* after overexpression of *TRA2B‐L* (left) or TRA2B‐S (right) (*n* = 3). (G, H) Analysis of the protein expression levels and grey value of *MyoD1*, *MyoG* and *MyHC* after overexpression of *TRA2B‐L* (G) and *TRA2B‐S* (H) (three biological replicates shown in blots; *n* = 3). (I) *MyHC* staining for analysing myotube after overexpression of indicated vectors in myoblasts which were induced into differentiation for 48 h. Fused myotubes were positive for MyHC (green), and cell nuclei were positive for DAPI (blue). Bar 150 μm. (J–L) *TRA2B‐L*, *TRA2B‐S* and pcDNA3.1 vectors were transfected into chicken myoblasts, and myotube formation and differentiation index were analysed. (M) mRNA abundance of *Ki‐67*, *PCNA* and *CCND1* after overexpression of *TRA2B‐L* (left) or *TRA2B‐S* (right) (*n* = 3). (N) Cell proliferation was assessed with CCK‐8 assay after overexpression of *TRA2B‐L* and *TRA2B‐S* (*n* = 6). (O) Cell proliferation was assessed with EdU after overexpression of *TRA2B‐L* and *TRA2B‐S*. Bar 150 μm. (p) The percentage of proliferative myoblasts after overexpression *TRA2B‐L*, *TRA2B‐S* and pcDNA3.1 (*n* = 6). (Q) Myoblasts were overexpressed with *TRA2B‐L*, *TRA2B‐S* and pcDNA3.1, and the cell cycle phase was then analysed (*n* = 4). (R) Representative photographs of H&E staining of gas muscle transfected with LV‐TRA2B‐L. (S) The mRNA expression of *Ki‐67*, *PCNA* and *CCND1* after overexpression of *TRA2B‐L* in gas muscle. (T) The mRNA expression of *MyoD1*, *Myogenin* and *MyHC* after overexpression of *TRA2B‐L* in gas muscle. (U) Comparison of the percentage of muscle fibres with the indicated cross‐sectional area (μm^2^) in LV‐TRA2B‐L and negative control. (V) Representative photographs of H&E staining of chicken gas muscle transfected with LV‐TRA2B‐S. (W) The mRNA expression of *Ki‐67*, *PCNA* and *CCND1* after overexpression of *TRA2B‐S* in gas muscle. (X) The mRNA expression of *MyoD1*, *Myogenin* and *MyHC* after overexpression of *TRA2B‐S* in gas muscle. (Y) Comparison of the percentage of muscle fibres with the indicated cross‐sectional area (μm^2^) in LV‐TRA2B‐S and negative control. All the values are mean ± SEM. The significance of each detected change between the two groups was evaluated by Student's *t* test. *p < 0.05, **p < 0.01 and ***p < 0.001. AS, alternative splicing; CCK‐8, cell counting kit‐8; H&E, haematoxylin and eosin.

Our analysis of AS in both ISO‐seq and scRNA‐seq data sets revealed significant AS events occurring in the TRA2B gene. However, the functional role of the TRA2B isoforms in myogenesis remains unknown. Next, we investigated the contribution of TRA2B isoforms on myogenesis. To investigate the contribution of TRA2B isoforms on myogenesis, we cloned Flag‐tag in‐frame with the N or C terminus of the TRA2B ORFs. Results showed that both N‐terminal and C‐terminal Flag‐tag isoforms can be encoded (Figure 5A). To further evaluate the coding potential of both transcripts, the ORFs of TRA2B transcripts were cloned to construct fusion proteins with GFP. The green signal verified that both *TRA2B‐L* and *TRA2B‐S* can be encoded into function proteins (Figure 5B). To determine the effect of any terminal signal in 5′UTR on the transcription of *TRA2B* transcripts, we created the expression vectors of fusion proteins with 5′UTR of each alternatively spliced isoform. The results showed that both 5′UTR of *TRA2B‐L* and *TRA2B‐S* did not affect the coding potential of downstream ORF (Figure 5B). Throughout embryonic muscle development, the expression of *TRA2B‐L* was relatively stable, while the expression of *TRA2B‐S* was substantially elevated during embryonic development (Figure 5C). Interestingly, *TRA2B* isoforms were highly expressed in striated muscle, with the highest expression in leg muscle (Figure 5D). We also examined the expression of both isoforms during myoblast differentiation. During myoblast differentiation, the expression of *TRA2B‐L* was downregulated and then upregulated, whereas *TRA2B‐S* significantly increased its expression (Figure 5E). These findings suggested that *TRA2B* isoforms may play an essential role in myogenesis.

Given that *TRA2B* isoforms were all expressed during myoblast proliferation and differentiation, we overexpressed the specific isoforms of *TRA2B* to determine their effect on the cellular processes. Overexpression of one isoform had no influence on the expression of the other (Figure EV 20A,B). After *TRA2B‐L* overexpression, the mRNA and protein expression of myoblast differentiation marker genes, including *MyoD1* and *MyHC*, were significantly downregulated, whereas overexpression of *TRA2B‐S* increased their expression (Figure 5F–H). After overexpression, immunofluorescence staining was performed to further determine the role of each isoform in the regulation of myotube formation and myoblast fusion. Overexpression of *TRA2B‐L* significantly inhibited myotube formation and decreased the proportion of myotubes with more than 10 nuclei, whereas overexpression of *TRA2B‐S* promoted myotube formation and increased the proportion of myotubes with more than 10 nuclei (Figure 5I–L). Assays for cell proliferation were carried out. After overexpression of both *TRA2B* isoforms, there was a significant increase in the expression of proliferation marker genes (Figure 5M). The cell counting kit‐8 (CCK‐8) assay and 5‐ethyl‐2′‐deoxyuridine (EdU) staining demonstrated that *TRA2B* isoforms overexpression significantly increased myoblast proliferation and EdU incorporation, respectively (Figure 5N–P). Flow cytometric analysis demonstrated that overexpression of either of the two *TRA2B* isoforms substantially increased the number of cells in the S phase and decreased the number of cells that entered the G0/G1 phase (Figure 5Q). In addition, flow cytometric analysis revealed that overexpression of either *TRA2B* isoform substantially diminished the number of apoptotic cells (Figure EV 20C,D). These results suggested that both *TRA2B* isoforms can increase myoblast viability and regulate myoblast differentiation and fusion in opposing ways.

To gain a deeper comprehension of the involvement of *TRA2B* AS isoforms in the process of muscle fibre formation in vivo, we generated a lentiviral vector for each *TRA2B* isoform with the intention of inducing overexpression of the respective protein isoforms in the gastrocnemius (GAS) muscle of chickens (Figure 5R,V and Figure EV 21A). Upon overexpression of *TRA2B‐L* and *TRA2B‐S* in GAS, the proliferation marker genes in myoblasts were notably induced (Figure 5S,W). Conversely, the expression trend of differentiation marker genes was observed to be in the opposite direction following overexpression (Figure 5T,X). The results obtained from the haematoxylin and eosin (H&E) staining of GAS sections indicated that the overexpression of *TRA2B‐L* and *TRA2B‐S* led to an increase in the proportion of larger myofibrer (Figure 5U,Y).

### 2.7 *TRA2B* isoforms are required for myoblast survival and proliferation

**FIGURE 6:**
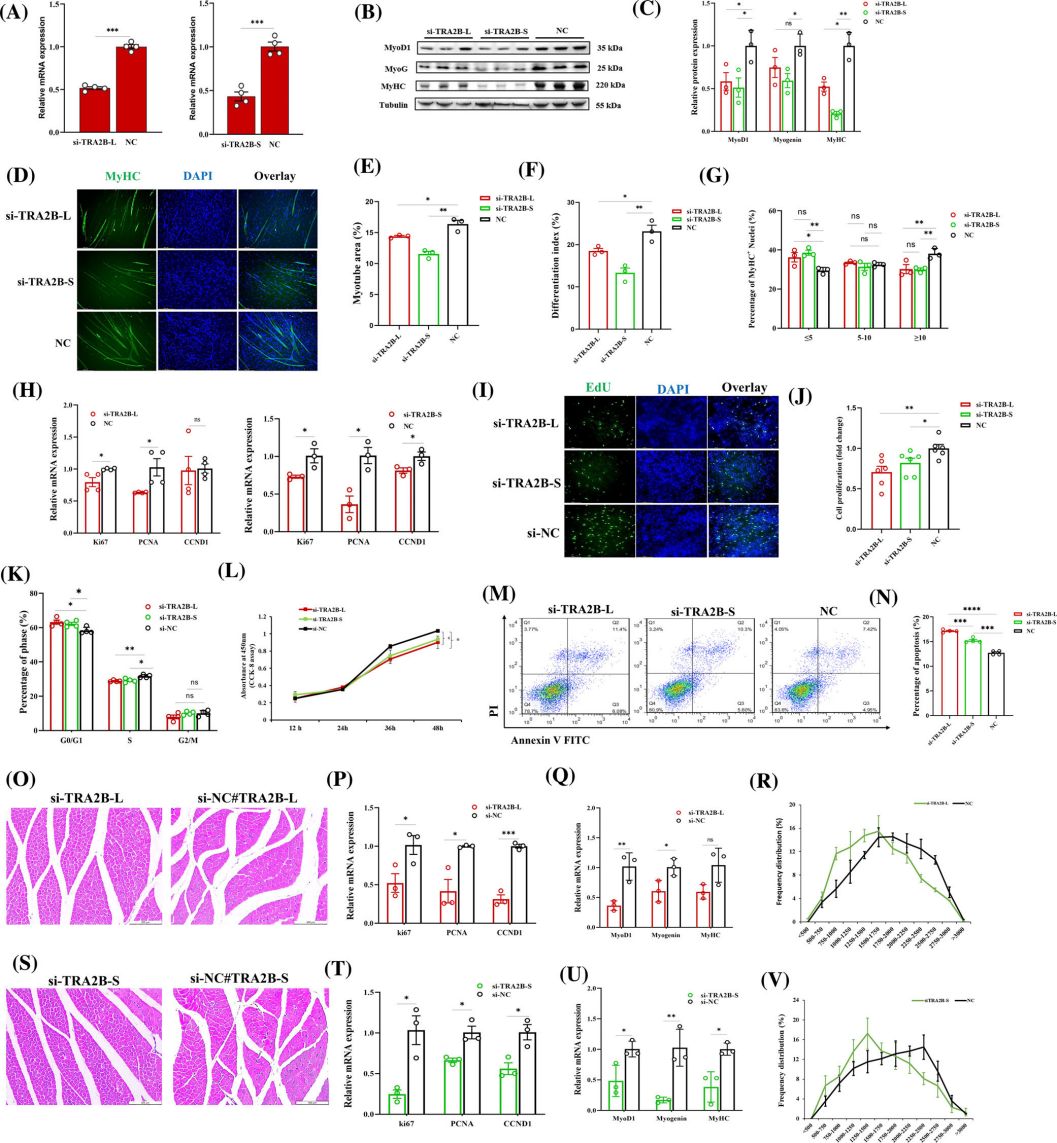
*TRA2B* isoforms are required for myoblast survival and proliferation. (A) qPCR analysis of the mRNA abundance of *TRA2B* and *TRA2B‐S* after transfection of specific small interfering RNA (siRNA) (*n* = 4). (B, C) Analysis of the protein expression levels and grey value of *MyoD1*, *MyoG* and *MyHC* after inhibition of *TRA2B* and *TRA2B‐S* (three biological replicates shown in blots; *n* = 3). (D) *MyHC* staining for analysing myotube after inhibition of *TRA2B* and *TRA2B‐S* in myoblasts which were induced into differentiation for 48 h. Fused myotubes were positive for MyHC (green), and cell nuclei were positive for DAPI (blue). Bar 150 μm. (E–G) Myotube formation and differentiation index were analysed after inhibition of *TRA2B* and *TRA2B‐S* (*n* = 3). (H) qPCR analysis of the mRNA abundance of *Ki‐67*, *PCNA* and *CCND1* after inhibition of *TRA2B* and *TRA2B‐S* (*n* = 3). (I) Cell proliferation was assessed with EdU after inhibition of *TRA2B* and *TRA2B‐S*. Bar 150 μm. (J) Percentage of proliferative myoblasts after inhibition of *TRA2B* and *TRA2B‐S* (*n* = 5). (K) Cell cycle phase was analysed after inhibition of *TRA2B* and *TRA2B‐S* (*n* = 4). (L) Cell proliferation was assessed with CCK‐8 assay after inhibition of *TRA2B* and *TRA2B‐S* (*n* = 6). (M, N) Cell apoptosis was determined by flow cytometry after inhibition of *TRA2B* and *TRA2B‐S* (*n* = 4; one‐way ANOVA with Tukey's multiple comparison test). (O) Representative photographs of H&E staining of gas muscle transfected with in‐vivo si‐TRA2B. (P) The mRNA expression of *Ki‐67*, *PCNA* and *CCND1* after inhibition of *TRA2B* in gas muscle. (Q) The mRNA expression of *MyoD1*, *Myogenin* and *MyHC* after inhibition of *TRA2B* in gas muscle. (R) Comparison of the percentage of muscle fibres with the indicated cross‐sectional area (μm^2^) in si‐TRA2B and negative control. (S) Representative photographs of H&E staining of gas muscle transfected with in‐vivo si‐TRA2B‐S. (T) The mRNA expression of *Ki‐67*, *PCNA* and *CCND1* after inhibition of *TRA2B‐S* in gas muscle. (U) The mRNA expression of *MyoD1*, *Myogenin* and *MyHC* after inhibition of *TRA2B‐S* in gas muscle. (V) Comparison of the percentage of muscle fibres with the indicated cross‐sectional area (μm^2^) in si‐TRA2B and negative control. All the values are mean ± SEM. Unless specified, the significance of each detected change between the two groups was evaluated by Student's *t* test. *p < 0.05, **p < 0.01, ***p < 0.001 and ****p < 0.0001. ANOVA, analysis of variance; CCK‐8, cell counting kit‐8; H&E, haematoxylin and eosin; qPCR, quantitative real‐time PCR.

Previous studies showed that the knockdown of *TRA2B* resulted in decreased cell viability in nerve cells,^31^, ^32^ so we sought to determine the effect of *TRA2B* isoforms knockdown on myoblast proliferation, cell viability and myotube formation. Two small interfering RNAs (siRNAs) targeting *TRA2B‐L* and *TRA2B‐S* were synthesised and transfected into myoblasts. The specific siRNA decreased the expression of both isoforms significantly (Figure 6A). *TRA2B‐S* knockdown substantially decreased the expression of myoblast differentiation marker genes. Meanwhile, *TRA2B‐L* knockdown decreased the expression tendency of *MyoD1*, *MyoG* and *MyHC* (Figure 6B,C). After *TRA2B* isoform reduction, myotube formation and myoblast differentiation were significantly suppressed (Figure 6D–F). In addition, suppression of *TRA2B* isoforms decreased the proportion of myotubes with more than 10 nuclei (Figure 6G). We questioned whether the suppression of *TRA2B* isoforms influences the proliferation and viability of myoblasts. The downregulation of proliferation marker genes, including *Ki67*, *PCNA* and *CCND1*, suggests that the suppression of *TRA2B* isoforms significantly reduced cell proliferation (Figure 6H). The EdU and CCK‐8 assays demonstrated that *TRA2B‐L* and *TRA2B‐S* interference significantly inhibited the proliferation of myoblasts (Figure 6I,J,L). After silencing *TRA2B* isoforms, the proportion of cells in the S phase decreased (Figure 6K). Furthermore, flow cytometric analysis revealed an increase in the number of apoptotic myoblasts following *TRA2B* isoforms knockdown (Figure 6M,N). In addition, in‐vivo siRNAs targeting *TRA2B‐L* and *TRA2B‐S* transcripts were designed to determine the effect of specific isoform silencing on postnatal myogenesis (Figure 6O,S). QPCR confirmed the inhibition of *TRA2B* isoforms (Figure EV 21B). Regardless of which isoform was silenced, proliferation and differentiation marker gene expression decreased substantially (Figure 6P,Q,T,U). After *TRA2B‐L* and *TRA2B‐S* suppression, the cross‐sectional area (CSA) of the GAS muscle decreased significantly, as evidenced by an increase in the proportion of smaller myofibres (Figure 6R,V), indicating that both isoforms are required for myogenesis.

### 2.8 *TRA2B* isoforms preferentially bind to primary mRNA exon regions to regulate splicing

**FIGURE 7:**
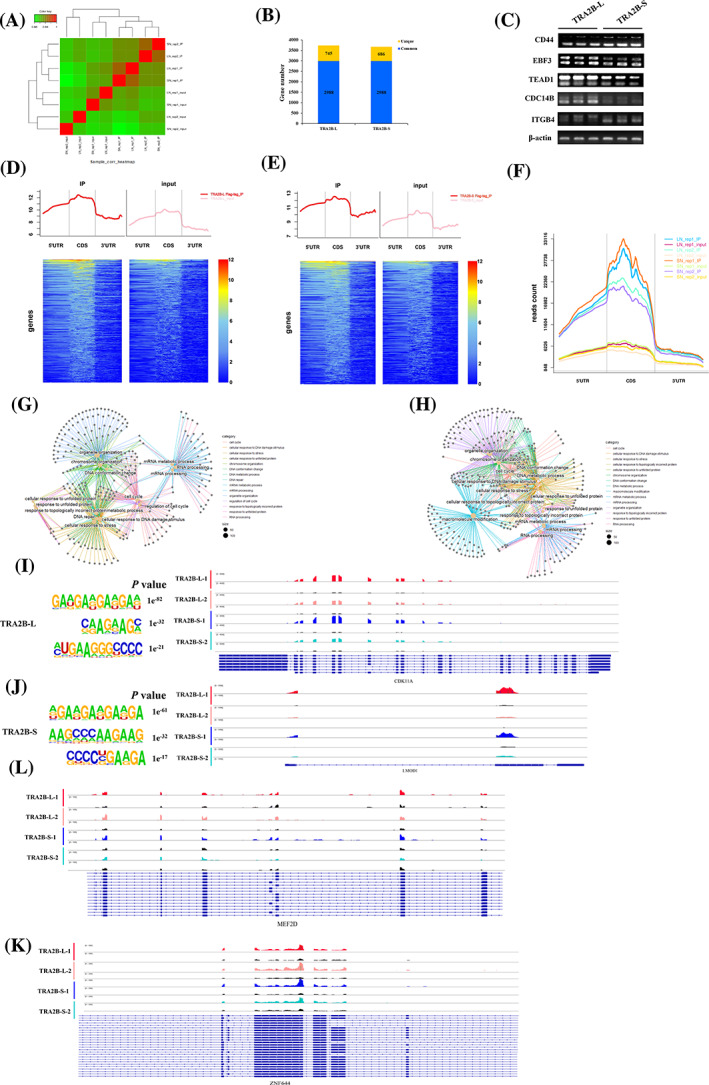
*TRA2B* isoforms preferentially bind to pre‐mRNA exon regions to regulate splicing. (A) Heatmap of Pearson's correlation coefficient between RIP‐seq from eight transcriptome libraries. (b) Common and specific targets of *TRA2B‐L* and *TRA2B‐S*. (C) RT‐PCR analysis of AS regulation of targets after overexpression of *TRA2B‐L* and *TRA2B‐S* in myoblasts. (D–F) Analysis of peaks enrichment in gene structural region of candidate targets of *TRA2B‐L* and *TRA2B‐S*. (G) Top biological function terms for genes combined by TRA2B‐L (targets were screened by a threshold of *p* value <0.001 and |log2FoldChange| > 1, compared to the input group). (H) Top biological function terms for genes combined by TRA2B‐S. (I) Transcript tracks of *CDK11A* and differentially enrichment region with read coverage, which is preferent combined by TRA2B‐L. The top three enriched consensus motifs for *TRA2B‐L* were shown in the left panel. (J) Transcript tracks of *LMOD1* and differentially enrichment region with read coverage, which is preferent combined by *TRA2B‐S*. The top three enriched consensus motifs for *TRA2B‐S* were shown in the left panel. (K) TRA2B‐L and TRA2B‐S exhibit position‐preferred exon‐binding properties on *ZNF644* transcripts. (L) TRA2B‐L and TRA2B‐S combined to the same region of alternative exon on *MEF2D*. AS, alternative splicing; RIP, RNA immunoprecipitation; RT‐qPCR, reverse transcription‐quantitative real‐time PCR.

To enhance comprehension of the AS transition mechanism facilitated by *TRA2B* isoforms in relation to myogenesis, we conducted RIP‐seq analysis on myoblasts that were subjected to transfection with Flag‐tag *TRA2B‐L* and Flag‐tag *TRA2B‐S*, respectively. RIP‐seq analysis showed that 3733 and 3674 genes were enriched in *TRA2B‐L* and *TRA2B‐S*, respectively (Figure 7A; Dataset EV 9). Of note, about 68% of genes can be immunoprecipitated by both isoforms of *TRA2B*, indicating that the *TRA2B‐L* and *TRA2B‐S* regulate AS of the same targets (Figure 7B). Five common targets obtained from RIP‐seq were used to verify the AS events regulated by *TRA2B‐L* and *TRA2B‐S*. The results showed that *TRA2B‐L* and *TRA2B‐S* play distinct effects on these AS (Figure 7C). Importantly, in both *TRA2B‐L* and *TRA2B‐S* immunoprecipitation tests, reads mapped in the reference genome were highly enriched in the CDS region (Figure 7D,E and Figure EV 22A–D). GO analysis revealed that TRA2B‐L‐targeted transcripts were mainly related to the regulation of protein structure, cell cycle and mRNA metabolic process (Figure 7G), while TRA2B‐S‐targeted transcripts were primarily associated with macromolecule modification, RNA processing, mRNA metabolic process, and cell cycle (Figure 7H). Homer de novo Motif Scan identified the common consensus motif (5′‐GAAGAAGAAG‐3′) was significantly enriched in the targeted region in *TRA2B* isoforms immunoprecipitations (Figure 7I,J). As shown in *CDK11A*, *LMOD1* and *ZNF644*, the coverage peaks of the targeted region showed that *TRA2B‐L* and *TRA2B‐S* bonded to target transcripts in the consensus motif‐dependent manner (Figure 7I–K). On the other hand, both isoforms of *TRA2B* bonded in the same region and further orchestrated the splicing outcome (Figure 7L). The above results showed that *TRA2B‐L* and *TRA2B‐S* bonded to the exons within the primary transcript to induce their constitutive splicing or regulate AS of the alternative exons in a position‐dependent manner.

### 2.9 The function of *TRA2B* isoforms on myoblast differentiation depends on their regulation of *TGFBR2* AS

**FIGURE 8:**
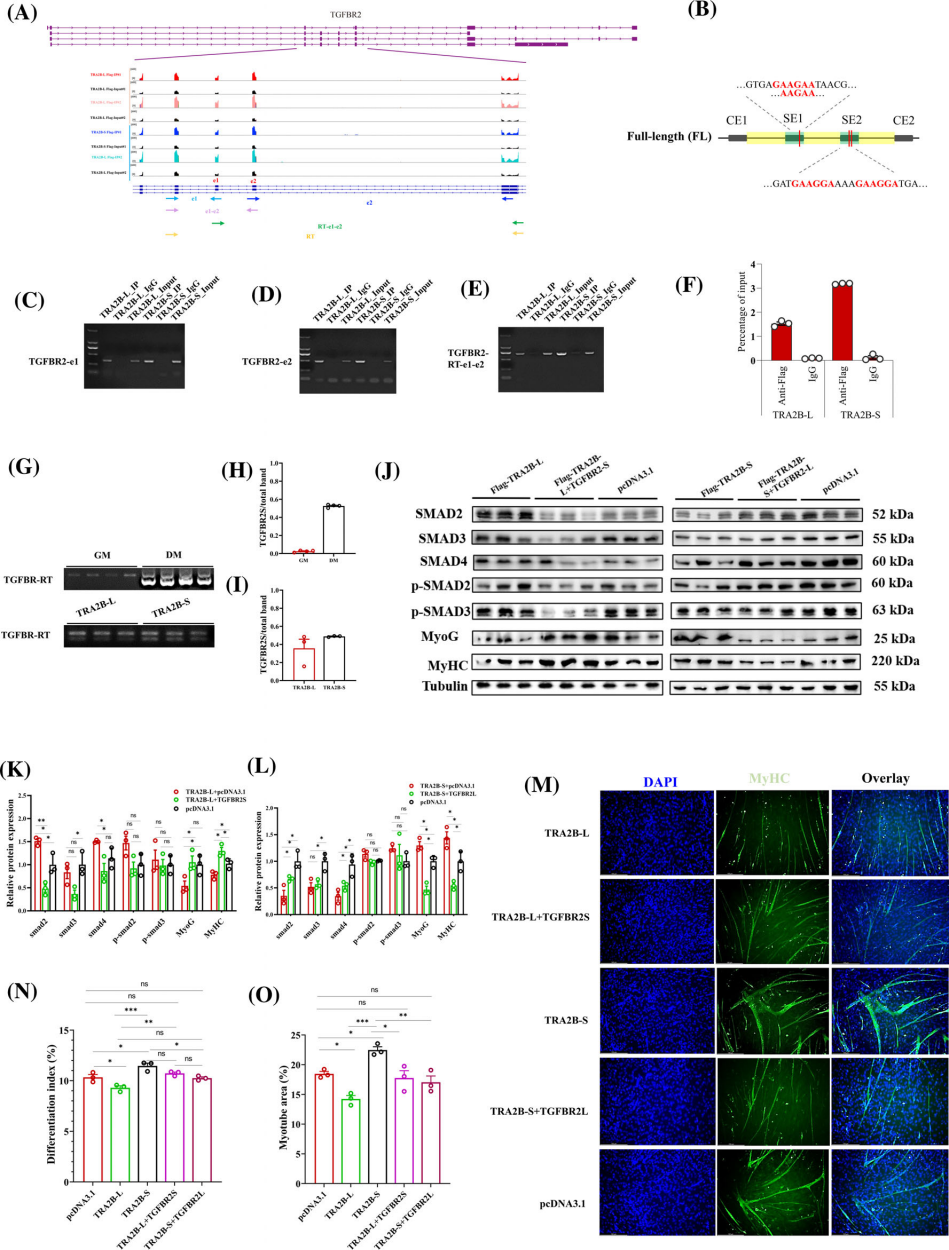
The function of *TRA2B* isoforms on myoblast differentiation depends on their regulation of *TGFBR2* alternative splicing. (A) Local AS of *TGFBR2*. The coloured arrow refers to the primer used for RIP‐PCR. (B) Screen of *TRA2B* motif at the alternative exons of *TGFBR2*. Redline marked the position of the *TRA2B* consensus motif. (C–E) RIP‐PCR results showed that exons e1 and e2 can bind to TRA2B‐L and TRA2B‐S. (F) Analysis of the grey value of RIP‐PCR using a primer of RT‐e1‐e2. (G–I) The splicing regulation of e1–e2 of *TGFBR2* during chicken myoblast differentiation and overexpression of *TRA2B‐L* and *TRA2B‐S*. (J) The protein levels and grey analysis of downstream of TGF‐β signalling pathway after overexpression of indicated overexpression vectors (three biological replicates shown in blots; *n* = 3). (M) MyHC staining for analysing myotube after transfected with indicated overexpression vectors in myoblasts which were induced into differentiation for 48 h. Fused myotubes were positive for MyHC (green), and cell nuclei were positive for DAPI (blue). Bar 150 μm. (N, O) Differentiation index and Myotube area of myoblasts that overexpression of indicated vectors. All the values are mean ± SEM. The significance of each detected change was evaluated by one‐way ANOVA with Tukey's multiple comparison test. *P < 0.05, **P < 0.01, ***P < 0.001. ANOVA, analysis of variance; AS, alternative splicing; RIP‐PCR, RNA immunoprecipitation‐polymerase chain reaction.

Since *TRA2B* isoforms play opposing roles in myoblasts differentiation, we hypothesised that *TRA2B* isoforms regulate AS by binding directly to a specific consensus motif within the same primary transcript region. To confirm that *TRA2B* isoforms regulate the same AS events, we scanned the differentially expressed peaks and DSGs between the two isoforms following the overexpression of specific isoforms in myoblasts. The *TGFBR2* gene's alternative exons e1 and e2 were evaluated as candidate regions bound by *TRA2B* isoforms (Figure 8A). SpliceAid and RBPmap identified an enriched motif within SE1, while two close consensus sequences appear in SE2 (Figure 8B; Dataset EV 10). We performed RIP‐PCR using specific primers targeting different regions to determine the direct combination between *TRA2B* isoforms and *TGFBR2* exons (Figure 8A). The results showed that both *TRA2B* isoforms combined with SE1 and SE2 of TGFBR2 transcript (Figure 8C–E). RIP‐qPCR also indicated that both regions could be immunoprecipitated by *TRA2B* isoforms (Figure 8F). To further validate the roles of *TRA2B* in *TGFBR2* isoform splicing, RT‐PCR was performed on chicken myoblasts in the GM and DM stages, as well as myoblasts transfected with *TRA2B‐L* or *TRA2B‐S* (Figure 8G). *TGFBR2S*, with SE1 and SE2 exclusion, was significantly induced in the DM stage. Interestingly, *TGFBR2S* increased its expression after overexpression of *TRA2B‐S* (Figure 8H,I). These results indicated that *TRA2B‐S* increased expression in the DM stage and therefore induced the exclusion of the alternative exons of *TGFBR2*. Since TGFBR2 can bind directly to TGF‐β ligands to activate canonical or noncanonical signalling pathways,^33^ we hypothesised that *TRA2B* isoforms could regulate canonical TGF‐β signalling via *TGFBR2*. Overexpression of *TRA2B‐L* significantly increased the expression of *Smad2*, *Smad3* and *Smad4*, indicating that the canonical TGF‐β signalling was activated, while overexpression of *TRAB‐S* significantly inhibited the canonical signalling (Figure 8J–L and Figure EV 23). Moreover, compared to the specific overexpression of *TRA2B‐L*, the expression *TGFBR2‐S* prevented the extensive activation of TGF‐β signalling by *TRA2B‐L*. The overexpression of the full‐length *TGFBR2* can reverse the inhibition of *TRA2B‐S* on TGF‐β downstream signal (Figure 8J–L). It is noteworthy that the phosphorylation of Smad2 and Smad3 remained unaltered subsequent to the overexpression of *TRA2B‐L* or *TRA2B‐S*, indicating that *TRA2B* isoforms regulated TGF‐β signalling by mediating the transition of *TGFBR2* isoforms (Figure 8J–L). Myosin immunofluorescence staining was performed to evaluate the effect of TGF‐β signalling mediated by *TGFBR2* on myogenesis. The results demonstrated that *TRA2B‐L* inhibited the formation and differentiation index of myotubes, whereas *TGFBR2S* reversed this effect. Alternatively, *TRA2B‐S* significantly accelerated myotube formation and increased the differentiation index, whereas overexpression of *TGFBR2* inhibited myotube formation induced by *TRA2B‐S* (Figure 8M–O). Collectively, *TRA2B* isoforms regulated the transition of *TGFBR2* isoforms during myogenesis.

## 3 DISCUSSION

NGS has an advantage in the estimation of gene expression, while it has a limited horizon to provide accurate transcript assembly. Characterising full‐length transcripts will facilitate functional genomics in certain biological processes, including myogenesis and embryogenesis. During the development of chicken myoblasts, we constructed a comprehensive transcriptome landscape using long‐read Iso‐Seq to characterise full‐length isoforms. We identified numerous full‐length isoforms among the expressed genes in primary myoblasts, proliferating myoblasts, and differentiated myotubes, of which many transcripts were not present in current genome annotations (Ensembl release 106). Our analysis of long‐read and short‐read RNA‐seq revealed that both transcriptional and post‐transcriptional transitions, particularly AS, are intimately associated with the alternation of the myogenic station. Although the structural categories and average length of full‐length isoforms are not substantially different between PM, GM, and DM myoblasts, the number of stage‐specific isoforms is significantly greater than the number of common transcripts. In addition, the majority of isoforms classified by SQANTI3 were identified as coding transcripts, indicating that these transcripts can translate into proteins or may have a regulatory function at the RNA level during myogenesis. We identified numerous fusion transcripts and alternative polyadenylation sites within a gene that underwent significant alterations during myoblast development using long‐read RNA‐seq data. Interestingly, some genes that have been confirmed to be associated with skeletal muscle function and structure exist in multiple AS forms, such as the TPM gene and the PDLIM7 family of genes, suggesting that genes expressed in skeletal muscle have a rich landscape of AS (Figure 1I and Figure EV 5D).^34^, ^35^, ^36^ Consequently, our data underscore the intricacy of the transcriptome during myogenesis and supports the notion that the structural characteristics of transcripts can be a source of tissue identity and phenotypic diversity.^37^

While formation of skeletal muscle relies on myogenic transcription factors that regulate gene expression at the transcription level, post‐transcription regulation provides another mechanism to affect myogenesis.^38^ AS modulates the diversity of the transcriptome and the proportion of alternatively spliced transcripts within a gene. To identify the repertoire of expressed isoforms during the development of chicken myoblasts, we combined the long‐read RNA‐seq data from PM, GM and DM samples to generate a master annotation. The master annotation revealed a total of 38,022 AS events, with SE and IR events accounting for the vast majority of AS categories. At each developmental stage, an estimated 30% of genes undergo AS (Figure 2B–D). Compared to a previous study that used splicing‐sensitive arrays to detect splicing transitions during C2C12 mouse myoblast differentiation, the proportion of SE events in this study is lower. This is primarily due to the difference in sensitivity of other AS events,^5^ particularly in the detection of alternative first exons.^39^ In contrast to IR transcripts, which are sensitive to the NMD pathway and dominant in plants,^40^ the majority of AS events during myogenic development are SE events,^41^ indicating that during skeletal muscle development, the majority of genes may produce distinct peptides by combining different exons within the same pre‐mRNA. Interestingly, the genes that undergo differential splicing transitions during myoblast differentiation are predominantly associated with the regulation of actin cytoskeleton, regulation of RNA splicing, membrane trafficking, regulation of the cell cycle, and function of skeletal muscle (Figure EV 8). The rearrangement of the actin cytoskeleton is essential for the function of multinucleated soma, which functions as a structural and supportive scaffold for sarcomeres and contributes to the plasticity of muscle.^42^ Notably, only a small number of genes that endure AS transitions during myoblast differentiation overlapped with genes whose expression levels varied during differentiation (Figure 2I). These findings support a regulatory mechanism involving splicing transitions from proliferative myoblasts to differentiated myotubes rather than a change in total gene expression.^41^

Outcomes of AS are intricately controlled by the RBPs and trans‐acting factors.^43^ RBPs play a crucial role in determining exon definition and splice site selection. Depending on where the RBPs bind in relation to the splice sites, the alternative region may be included or excluded from the resulting mature RNA transcripts.^16^ In a given cell type, multiple RBPs collectively regulate the distribution of alternatively spliced products for each AS event. During myogenesis, RBPs and RNA targets are expressed in a stage‐specific manner, making the decoding of regulatory information on indicated RNA targets highly dependent on the developmental stage.^17^ In this study, we systematically analysed the stage‐dependent expression variation of RBPs with identified consensus motifs in humans or mice (Figure 2K).^44^ Interestingly, several RBPs regulated opposing splicing directions in distinct stages (Figure 2L,M). SRSF10, which is believed to regulate myoblast differentiation by regulating the inclusion of alternative exons 16 and 17 of Lrrfip1,^45^ was significantly enriched in alternative exons by binding to the specific motif. During myoblast proliferation, SRSF10 increased the inclusion level of alternative exons, whereas it was highly enriched in downregulated exons during myoblast differentiation. Other RBPs, such as *QKI* ^46^ and *RBM24*,^47^ were also referred to as master regulators for muscle development by regulating the AS transitions. Our results demonstrated that *QKI* and *RBM24* regulate AS processes via a motif flanking omitted exons, and we determined that the regulatory phase of *QKI* and *RBM24* reversed from myoblast proliferation to differentiation.

Our scRNA‐seq analysis provided a comprehensive resource of myogenic differentiation in embryos. We identified seven cell types in cultured cells isolated from embryonic muscle, including muscle cells, macrophage cells, neurons, blood cells, fibroblasts, epithelial and endothelial cells. While previous studies have uncovered cellular heterogeneity, the majority of them have focused on samples collected from adult animals^48^, ^49^ or samples with a limited number of myogenic cells.^50^ Those resulted in insufficient cell populations to study cellular heterogeneity in myogenic differentiation. Similar to the DEGs identified in RNA‐seq, the highly expressed genes in muscle cells were mainly enriched in muscle development. Importantly, muscle cell was subset into eight cell types arranged from muscle progenitor to myofibre (Figure 3F). Furthermore, in this study, three kinds of myoblast exhibit distinct proliferative capacities and differentiation potential. Therefore, the cellular heterogeneity during embryonic development was explored in our scRNA‐seq, especially single‐cell populations like myoblasts.

Based on the current cell annotation results, we can trace the single‐cell transcriptome information of specific cell types and further analyse the splicing and gene expression differences between cell subpopulations. Two computational pipelines were used to determine the extent of splicing changes between cell types. Using splice‐aware peak calling to identify the relative peak usage can define several splicing events between cell types, including differential exon usage, alternative 3′UTR, alternative 5′UTR and intron retention.^23^ In the pairwise comparison of different cell types, the number of DTU peaks was the largest between muscle cells and macrophages, followed by the comparison group between fibroblasts and muscle cells (Figure 4A). In addition, DTU testing between myogenic differentiation indicated that many DTU peaks in cells at the end of differentiation and cells at the beginning of differentiation, suggesting that distinct cellular identities are associated with AS states.^37^ Another strategy to detect splicing dynamics in this study is differential splice junction‐based analysis, which calculated the PSI value to evaluate the splicing quantification, including SE, RI, A5SS, and A3SS. Consistent with results from the Sierra pipeline, the DSGs detected by the splice junction‐based approach were enriched in RNA metabolism, muscle differentiation, and actin filament‐based process (Figure 4J). Interestingly, contractile genes *TPM1*, *TPM2* and *TPM3* were differentially spliced across muscle subclusters (Figure 4K). Previous studies have reported that mutations in *TPM2* gene can cause abnormalities in muscle development and function, and abnormal transcript expression caused by splice site variations can also lead to muscle diseases.^51^, ^52^, ^53^ The structure of microtubules is closely related to cell function. During the differentiation and maturation of muscle cells, the fusion between single nuclei cells and changes in cell morphology require adaptive changes of microtubules.^54^, ^55^ We reasoned that regulation of AS of cytoskeleton‐related genes is involved in these processes. Gene‐splicing dynamics analysis of the comparison between muscle subclusters showed that most genes were subjected to differentially spliced without differentially expressed. This result confirms that in myogenic differentiation, splicing regulation provides the transcriptome with additional information beyond differential gene expression.

*TRA2B*, also known as *SFRS10*, is a member of the SR protein family with a unique structure. Its RRM domain is located in the middle of two RS domains. The two RS (arginine‐serine) domains of TRA2B protein can mediate the binding of TRA2B with other SR proteins and bind with RNA to promote RNA–RNA pairing. Besides, the RRM domain can specifically recognise the 5′‐ AGAA‐3′ motif in pre‐RNA.^56^ The specific knockout of *TRA2B* in the cerebral cortex leads to neuronal apoptosis and cortical structural abnormalities, indicating that TRA2B is indispensable for neuronal survival.^31^ Decreased TRA2B expression was found in obese humans and high‐fat feeding‐induced obese mice, resulting in increased expression of the lipid‐regulating gene *LIPIN1β* isoform, promoting adipogenesis and lipid accumulation.^57^ Specific knockdown of *TRA2B* in the toad (Xenopus) resulted in abnormal somit development due to misregulation of AS events. Notably, *WNT11b*, a Wnt ligand, retained the last intron after *TRA2B* knockdown, resulting in a truncated protein and suppressing full‐length transcript expression.^58^ These studies suggest that *TRA2B* may affect cellular processes by activating constitutional splicing or regulating AS of target transcripts dependent on the cell type or tissue background. Interestingly, we noticed that an isoform increased its expression during myoblast differentiation. Meanwhile, coding potential analysis confirmed that the exon inclusion of *TRA2B* pre‐mRNA can produce a truncated protein without the first RS domain. The two RS domains interact with other RS‐containing proteins or with each other or interact with RNA to maintain the RNA–RNA base pairing.^59^ Therefore, the lack of RS1 may disrupt the interaction of RNA–RNA pairing protein binding. We investigated the biological distinction between *TRA2B‐L* and *TRA2B‐S* with regard to myogenesis. *TRA2B‐L* and *TRA2B‐S* were highly expressed in breast and leg muscles, respectively. And *TRA2B‐S* expression increased substantially during myogenic differentiation, whereas *TRA2B‐L* expression remained relatively constant. Additional research revealed that both *TRA2B* isoforms are required for myoblast viability, proliferation and myofibre formation. Of note, *TRA2B‐S* induced myogenic differentiation, while *TRA2B‐L* suppressed the myotube formation and differentiation. Previous studies have shown that RBPs play a crucial and necessary role in maintaining postnatal muscle physiology.^47^, ^60^ However, the impact of *TRA2B* AS transcripts on the physiological functions of postnatal or adult muscles, such as grip strength, physical strength, or regeneration performance, remains unknown. Understanding the broader physiological functions of *TRA2B* AS transcripts in muscle will help elucidate the mechanisms that regulate muscle development and functional maintenance. RIP‐seq results showed that most of the RNA targets of *TRA2B‐L* and *TRA2B‐S* are common, and these target genes are mainly involved in the cell cycle and RNA metabolism. Importantly, the central 5′‐AGAA‐3′ motif within alternative exons is recognised by *TRA2B‐L* and *TRA2B‐S* via sequence affinity of the RRM domain. We proposed that the alteration of relative expression of *TRA2B* isoforms directed the AS of targets to regulate myogenic differentiation.

In our study, we have identified *TGFBR2* as a critical splicing target of *TRA2B* isoforms in chicken primary myoblasts. Interestingly, we observed that two alternative exons of *TGFBR2*, namely e1 and e2, had an increased proportion of skipped transcripts during myoblast differentiation. Conversely, the expression of the full‐length transcript showed a slight increase (as shown in Figure 8G). Canonical TGF‐β signalling is activated by when one of the TGF‐β1, 2 and 3 ligands binds to TGFBR2, which then recruits and phosphorylates TGFBR1 and further phosphorylates Smad2 and Smad3.^33^ Therefore, TGFBR2 acts as a gatekeeper for TGF‐β signalling for the activation of downstream signalling. TGF‐β signalling plays an inhibitory role in myogenesis. It was reported that TGF‐β signalling effector Smad3 represses *Myogenin* expression and interacts with *MEF2C* to disrupt the interaction between *MEF2C* and SRC‐family coactivator GRIP‐1, thus diminishing the transcription activity of *MEF2C*.^61^ During mouse C2C12 myoblast differentiation, miR‐122 suppresses the TGF‐β/SMAD signalling by directly targeting *TGFBR2* to restore myogenesis.^62^ Here, we presented that *TRA2B* isoforms regulated the position‐dependent AS of e1 and e2 exons of *TGFBR2* and, consequently, the modulated activity of TGF‐β/SMAD signalling. Notably, the expression of the truncated transcript of *TGFBR2* (namely *TGFBR2S*) partly rescued the inhibitory roles of *TRA2B‐L* on myotube formation, while the full‐length of *TGFBR2* reduced the extensive effect of *TRA2B‐L* on myoblast differentiation. We hypothesised that *TGFBR2* isoforms can interact in a concentration‐dependent fashion. We cannot, however, rule out the possibility that additional RNA targets regulated by *TRA2B* influenced myogenic differentiation. We investigated the interaction between TRA2B‐S and TGFBR2‐S/TGFBR2‐L and its potential role in signalling regulation. Further researches are needed to identify these domains and validate their functional relevance. Additionally, other proteins may assist TRA2B‐S in regulating target gene splicing. Co‐IP experiments can identify these proteins and elucidate their roles in splicing regulation. As multiple RBPs coordinated the splicing consequence of a given RNA target, it will be interesting to determine how the splicing network involving TRA2B isoforms and other splicing factors operates.

## 4 MATERIALS AND METHODS

### 4.1 Animal and tissue preparation

Xinhua female chickens were obtained from the South China Agricultural University farm. All tissue samples were collected and stored at −80°C. The sex of each embryo was determined by PCR using sex‐specific primers as previously described.^63^ The protocols of the animal experiments were approved by the South China Agricultural University Institutional Animal Care and Use Committee (Permission: SACU‐2018f052). And the methods were carried out in accordance with the regulations and guidelines established by this committee.

### 4.2 Cell culture

As previously described,^64^, ^65^ chicken myoblasts were isolated from chicken leg and breast muscle, which were classified into PM (primary myoblasts, which were collected after the second round of plating), GM and DM according to culture time and differentiation status. GMs were cultured in growth medium with RPMI‐1640 (Gibco), 20% fetal bovine serum (FBS) (Gibco), 10% chicken embryo extract and 0.2% penicillin/streptomycin (Gibco) at 37°C in 5% CO_2_. When the density of GM‐phase myoblasts reached 90%, the medium was changed to the differentiation medium (which removes FBS from GM medium) to induce differentiation for 4 days (DMs).

### 4.3 10× genomics single‐cell library preparation

The PM, GM and DM cells were transferred to the 10× Genomics Single Cell 3′ Chip according to the standard protocol. The libraries from each sample were sequenced using Illumina Novaseq 6000 with a paired‐end 150 bp reading strategy by Genedenovo Biotechnology Co., Ltd (Guangzhou, China).

### 4.4 Raw data analysis of scRNA‐seq

The CellRanger (V7.0.1) was used to demultiplex Illumina BCL results into fastq format. The alignment of reads onto the chicken GRCg6a.104 reference genome was created by the CellRanger count function. Doublets of each sample were detected and removed using DoubleFinder.^66^ Seurat package (V4.3.0) was used for downstream analysis.^67^ In brief, the cells that meet the conditions: (i) >3500 or <200 genes, (ii) mitochondrial percentage >15% were removed. The expression matrix was normalised with the ‘NormalizeData’ function of Seurat, with the ‘LogNormalize’ function and a scale factor of 1e4. The expression data were centred and scaled using the ‘ScaleData’ after the ‘FindVariableGenes’ function was performed with the parameters: selection.method = ‘vst’ and nfeatures = 2000. ‘SCTransform’ function was used to remove batch effect.^68^ Briefly, each Seurat object from PM, GM and DM was transfected into ‘SCTransform’ with the parameter: variable.features.n = 3000. After that, the functions of ‘SelectIntegrationFeatures’, ‘PrepSCTIntegration’, ‘FindIntegrationAnchors’ and ‘IntegrateData’ were performed using the default parameters. After batch correction, principal component analysis (PCA) was conducted with ‘RunPCA’ function. The UMAP dimensional reduction based on the first 30 principal components was performed using the ‘RunUMAP’ function. A shared nearest neighbour graph was created using the first 30 principal components using ‘FindNeighbours’ function. The cells with similar expressions were clustered using the ‘FindClusters’ function with the 0.1 resolution.

### 4.5 Cell type annotation and marker identification

DEGs for each identified cluster were screened using the ‘FindAllMarkers’ with Wilcox test. A gene considered as DEG must be expressed in 25% of cells and exhibit logFoldChange of at least 0.25. The top 20 DEGs were used to visualise. Cell‐type‐specific genes were identified using the criterion with logFC > 1 and adjusted *p* < 0.05. The heatmaps showing the gene expression across cell types were performed using scRNAtoolVis (https://github.com/junjunlab/scRNAtoolVis). Annotation of cell types was analysed based on the expression of canonical markers for particular cell types.^69^

### 4.6 Pseudotime trajectory analysis

To determine the potential lineage differentiation, we applied the Monocle3 (V1.0.1),^70^ and scVelo (V 0.2.3) with the dynamical model.^71^

### 4.7 Detecting differential peak usage

We applied Sierra R package to calculate differential peak usage^23^ with minor modifications. In brief, a merged BAM file from each sample produced by CellRanger was used to perform splice‐aware peak calling. Splice junctions file was generated by the ‘junctions’ function of regtools (V0.5.2). The peaks were counted by ‘CountPeaks’ function using the set of unified peak coordinates from peak coordinates file. The peaks were annotated using GTF and BSgenome.Ggallus.UCSC.galGal6, and further identified the boundaries within the known genes (UTR, exon and intron). DTU was tested using adapted DEXSeq (V1.24.4). Differential usage peaks were identified according to an adjusted *p* value <0.05 and |logFC| > 0.25.

### 4.8 Splice junctions and gene‐splicing dynamics analysis

To detect splice junctions between cell types, the barcodes of each cell type from annotated Seurat object were used to retrieve the merge BAM file using subset‐bam tool (V1.1.0) (https://github.com/10XGenomics/subset-bam). The extracted BAM file of each cell type was used to produce gene and splice junction count matrices using STARsolo (V2.7.8a). The output of STARsolo was transferred to SingCellaR (V1.2.1) to perform integration of gene expression, cell metadata and gene metadata, which is required for downstream processing.^72^ Raw gene count and splice junction count of each cell type generated by STARsolo were merged, respectively. The resulting output (gene counts, splice junction counts and normalised gene expression) was used to create MARVEL (V1.4.0) object according to recommended instructions (https://wenweixiong.github.io/MARVEL_Droplet.html#Gene‐splicing_dynamics). To investigate the correlation between gene expression and splicing, MARVEL classified gene‐splicing dynamics into coordinated, opposing, isoform‐switch and complex, which was assigned by ‘IsoSwitch.10X’ function with default parameters.

### 4.9 Total RNA extraction, first‐strand cDNA synthesis, reverse transcription and quantitative real‐time polymerase chain reaction

Total RNA was extracted from all samples or cells using TRIzol:chloroform RNA extraction according to the manufacturer's recommended protocols (Invitrogen). The first‐strand cDNA synthesis was carried out with PrimeScript™ RT reagent Kit (Takara) according to the manufacturer's manual. Quantitative real‐time polymerase chain reaction (qPCR) was performed with ABI QuanStudio 5 qPCR System (Applied Biosystem Inc.), following the method as described.^73^ Standard RT‐PCR was performed to validate AS using primers that flank specific alternative exons, and PCR products were separated on a 1.25% agarose gel. The gel was imaged and the PSI for evaluating the level of splicing transition between different situations was calculated as previously described.^60^ All specific mRNA PCR primers were provided in Dataset EV 1.

### 4.10 Short‐read and long‐read library construction and sequencing

For short‐read RNA‐seq, the chicken PM, GM and DM cells were harvested and total RNA was extracted using RNAiso reagent (Takara) following the protocol provided by the manufacturer. The poly(A) RNA was isolated for paired‐end 100‐bp sequencing using Illumina HiSeq 2000 platform (Illumina). To determine RNA quality, samples from the three biological replicates of each phase were assessed using a NanoDrop microspectrophotometer (Thermo Fisher Scientific, https://www.thermofisher.com/) and an Agilent 2100 Bioanalyzer (Agilent Technologies, https://www.agilent.com/). Raw read quality was assessed using the FastQC suite version 0.10.1. RNA‐seq reads were aligned to the chicken genome (*Gallus gallus* build 6.0 from ENSEMBL) using HISAT 2.0.4 with default parameters. HTSeq V0.6.1 evaluated the expression level of genes annotated by ENSEMBL with ‐m union. Each gene's FPKM (fragments per kilobase of exon per million mapped fragments) and TPM (transcripts per kilobase million) value were transformed from raw read count using custom script. The DEGs were identified with DESeq using the criteria *p*adj < 0.05 and |log2FC| ≥ 2.

For long‐read RNA‐seq, only samples with RIN 8.0 and greater were selected for library preparation. The iso‐seq library was prepared according to the Isoform Sequencing protocol (Iso‐Seq) using the Clontech SMARTer PCR cDNA Synthesis Kit for first‐strand cDNA synthesis and the BluePippin Size Selection System protocols described by Pacific Biosciences (PN 100‐092‐800‐03). A total of 12 PCR cycles of amplification were performed for each sample. PacBio Sequal System was used for sequencing, and four cells were run with each sample for movie run‐time of 600 min.

### 4.11 RNA immunoprecipitation and sequencing

The chicken primary myoblasts were transfected with flag‐tag TRA2B‐L and TRA2B‐S overexpression vectors for 48 h, respectively. Then, the myoblasts were harvested for RNA immunoprecipitation (RIP). The transfected myoblasts were lysed in cold lysis buffer with 50 mM Tris, 150 mM NaCl, 2 mM ethylenediaminetetraacetic acid (EDTA), 0.1% sodium dodecyl sulphate (SDS), 0.5% NP‐40, 0.5% deoxycholate, 1 mM dithiothreitol, 200 U/mL RNase inhibitor and protease inhibitor cocktail for 1 h on ice. For a better lysis effect, mix the lysis mixture every 5 minutes. The supernatant was collected and incubated with 10 μg of anti‐Flag antibody (bsm‐33346, Bioss) and anti‐IgG (bs‐0310P, Bioss) overnight at 4°C. Adding protein A Dynabeads to supernatants for another 4 h incubation. The beads were enriched using a magnet. And the beads were washed two times with lysis buffer, high‐salt buffer (250 mM Tris 7.4, 750 mM NaCl, 10 mM EDTA, 0.1% SDS, 0.5% NP‐40 and 0.5 mM deoxycholate) and PNK buffer (50 mM Tris, 20 mM ethylene glycol tetraacetic acid and 0.5% NP‐40) as previously described with minor modification.^74^ The RNAs were extracted from the beads with elution buffer and then were reverse‐transcribed into cDNA. The libraries were prepared according to the manufacturer's recommended protocol on an Illumina HiSeq 2000 platform.

### 4.12 Isoform identification and structural classification

The PM, GM and DM cells produced a total of 61 gigabytes of long‐read RNA transcriptome data. The raw subreads were analysed following the SMRTlink 5.1.0. CCS.bam was generated from subreads.bam with the following parameters: ‐‐minLength 200 ‐‐maxDropFraction 0.8 –minZScore ‐9999 –minPasses 1 –minPredictedAccuracy 0.8 –maxLengths 18000. The primer fastas were set to: F0 AAGCAGTGGTATCAACGCAGAGTACATGGG; R0 GTACTCTGCGTTGATACCACTGCTT. And all CCS was classified into full‐length non‐chimera (FLNC) and non‐full‐length (nFL). Full‐length reads were clustered and polished using pbtranscript cluster with the following key parameters: ‐‐hq_quiver_min_acuracy 0.99 –qv_trim_5 100 –qv_trim_3 30. The clustered and polished reads were corrected using Illumina RNA‐seq data with LoRDEC 0.9 using the following parameters: ‐k 19 ‐s 3. The corrected effect was evaluated using lordec‐stats. The corrected reads were aligned to the Gallus_gallus.GRCg6a (Ensembl build 6.0) using minimap2 (v2.13) with the following parameters: ‐ax splice ‐uf –secondary=no ‐C5 ‐O6,24 ‐B4.^75^ Redundant isoforms were collapsed and then the abundance file of each full‐length isoform using cDNA_Cupcake collapse scripts and get_abundance_post_collapse.py according to the recommended parameters (https://github.com/Magdoll/cDNA_Cupcake/wiki). To create a master transcriptome for myoblasts proliferation and differentiation profile, abundance files and aligned reads for each phase were chained using cDNA_Cupcake chain_sample.py. Characterisation of corrected isoforms was performed by SQANTI3.^76^ SQANTI catalogued isoforms into nine different descriptors. Isoforms were filtered using sqanti_filter.py and the filtered data set was run again with sqanti_qc.py as previously described.^75^

### 4.13 Identification and clustering of AS events and differential expression isoforms

To detect AS events and calculate the PSI value for each AS event using short‐read RNA‐seq, rMATS was used as previously described.^77^ The output files of rMATS were conveyed to RMAPS to map and plot the binding sites of RBPs using default parameters.^78^ For Iso‐Seq, AS events were generated using SUPPA.^79^ The PSI value for a local event or a transcripts isoform was calculated using suppa.py diffSplice with default parameters. The AS events change between PM, GM and DM at similar behaviours were clustered using density‐based clustering with parameters: ‐e0.05 ‐n 10 ‐dt 0.05 ‐st 0.05 ‐c OPTICS. The differentially expressed isoforms with similar change using R package MFUZZ.^80^ The 200 bp sequence of upstream and downstream intron flanking alternative exon was extracted using bedtools (version 2.30.0), and the enriched motifs were analysed modified from the previous study using HOMER findMotif.pl script with parameters: ‐len 6,8,10,12.^81^ The significantly AS events were selected as regulated events, while the other events were selected as background events.

### 4.14 Functional enrichment analysis

GO and Kyoto Encyclopedia of Genes and Genomes analyses were carried out with ClusterProfilter.^82^ Functional enrichment was performed using the web‐based Metascape database.^83^ Gene set enrichment analysis for scRNA‐seq was performed using GSVA R package (V1.42.0) using the hallmark gene set retrieved from msigdbr.^84^

### 4.15 Immunoblotting and immunofluorescence

Western blot was as previously described.^85^ Primary antibodies used here were anti‐Flag (1:1000, bsm‐33346, Bioss), anti‐MyoD (1:1000, bs‐2442R, Bioss), anti‐MyoG (1:1000, bs‐3550R, Bioss), anti‐MyHC (1:300, B103, DSHB), anti‐Tubulin (1:1000, bs‐0715R, Bioss), anti‐SMAD2 (1:1000, bs‐0718R, Bioss), anti‐SMAD3 (1:1000, bs‐3484R, Bioss), anti‐SMAD4 (bs‐23966R, Bioss), anti‐p‐SMAD2 (detection of Ser 465 and Ser467 phosphorylated SMAD2. 1:1000, bs‐3419R, Bioss) and anti‐p‐SMAD3 (detection of Ser423 and Ser425 phosphorylated SMAD3. 1:1000, bs‐3425R, Bioss). The immunofluorescence was performed using anti‐MyHC (1:50, B103, DSHB) and DAPI (Beyotime) as previously described.^85^, ^86^ The cells were fixed with a 4% formaldehyde solution for a duration of 20 min, followed by three washes with PBS for 5 min each time. Subsequently, cell permeabilisation was achieved by treating with 0.1% Triton X‐100 for 15 min. To prevent non‐specific binding, the cells were then blocked using goat serum for 1 h. Following an overnight incubation at 4°C with an anti‐MyHC antibody, the cells were exposed to fluorescein isothiocyanate (FITC)‐conjugated AffiniPure Goat Anti‐Mouse IgG (H + L) (1:100; BS50590, Bioworld) and incubated in darkness for 1 h. The total myotube area was calculated as the percentage of the total image area covered by the myotube, while the differentiation index represented the number of nuclei inside the MyHC‐positive myotubes relative to the total number of nuclei. A Nikon Eclipse TieU fluorescence microscope was used to capture fluorescence images. In each well, five fields of view were randomly selected and analysed. The nuclei number was calculated using ImageJ software (National Institutes of Health). The immunofluorescence results were shown from three biologically independent experiments.

### 4.16 Plasmid construction, RNA oligonucleotides synthesis and cell transfection

The coding sequences of chicken TRA2B‐L and truncated TRA2B‐S were amplified using specific primers and cloned into pcDNA3.1 (Invitrogen) (Dataset EV 1). siRNAs targeting specific regions for *TRA2B* isoforms are produced by HanBio, and a non‐specific duplex was used as the control. Lipofectamine 3000 reagent (Invitrogen) was used to perform transfection. The working concentration of siRNA is 100 nM. The procedure of transfection was performed following the manufacturer's protocol. The in vivo interference experiment was slightly modified based on previous research.^87^, ^88^ The in‐vivo siRNA (RiboBio) was injected at a concentration of 20 μmol. The injection was performed once every 3 days. For each chick, the gastrocnemius muscle of the left leg was the experimental group and the gastrocnemius muscle of the right leg was the control group (*n* = 6). Samples were collected at 14 days after the first injection.

### 4.17 Lentivirus production and transduction

A mixture of pWPXL overexpression vector (pWPXL‐3xFlag‐TRA2B‐L, pWPXL‐3xFlag‐TRA2B‐S or pWPXL‐3xFlag‐GFP), psPAX2 and pMD2.G were transfected into HEK293T cells using Lipofectamin 3000 reagent to generate lentivirus. The supernatants were collected 72 h later and filtered through 0.45 μm PVDF membranes (Millipore) and cleared by supercentrifugation. The viral titre was evaluated by a gradient dilution. Chicks at the indicated days were infected with lentiviruses (1 × 10^8^ infection unit per chick, *n* = 6) by direct injecting into the gastrocnemius muscle as previously described.^85^

### Cell viability assays

For CCK‐8 assays, the myoblasts cultured in a 96‐well plate were transfected, and the cell viability was determined at 12, 24, 36 and 48 h using the CCK‐8 reagent (HB‐CCK8‐3, HanBio). A total of 10% CCK solution was added to the medium and incubated for 1 h. Model 680 Microplate Reader (Bio‐Rad Laboratories Inc.) was used to measure the absorbance at 450 nm. For flow cytometric analysis, the myoblasts that have been transfected for 48 h were fixed in 70% ethanol overnight at 4°C. The cells were treated with PI/RNase staining buffer (Beyotime) according to the product manual. Cell proliferation was calculated using a CytoFLEX flow cytometer (Beckman Coulter, S. Kraemer Boulevard Brea) and analysed using ModFit Lt 4.1 software (Verity Software House). Cell apoptosis was evaluated after staining by an annexin V‐FITC/propidium iodide (PI) staining detection kit (Beyotime) according to the manufacturer's instructions. For EdU assay, the myoblasts that have been transfected for 48 h were incubated for 2 h in 50 μM 5‐ethynyl‐2′‐deoxyuridine (RiboBio). And the cells were fixed in 4% paraformaldehyde for 20 min and stained with EdU Apollo in Vitro Imaging Kit (RiboBio). After EdU staining, the cells were observed under a fluorescence microscope (Nikon). Five randomly selected fields of each well were used to calculate the ratio of positive cells to all myoblasts.

### 4.19 Histology and muscle phenotype measurements

The skeletal muscle samples were collected at an indicated time and fixed with 4% paraformaldehyde. Fixed skeletal muscles were paraffin‐embedded, sectioned and stained with H&E. Images were captured using an optical microscope (Leica). The CSA of each muscle fibre for each chick was analysed. At least five randomly selected non‐overlapping images were used to evaluate the effect on muscle fibre development.

### 4.20 Statistical analysis

The statistical significance of multiple groups was determined by one‐way or two‐way analysis of variance with Tukey's multiple comparison test in mean value at the 5% significance level. Two‐sided Student's *t* test was used to determine the statistical significance of the two groups. We used GraphPad Prism 9.4.1 software to plot data in mean values, with error bars representing the standard error of mean. All experiments were performed at least three biological times.

## AUTHOR CONTRIBUTIONS

Wen Luo, Xiquan Zhang and Qinghua Nie conceived the project. Wen Luo and Genghua Chen designed the experiments. Genghua Chen, Jiahui Chen, Lin Qi, Yunqian Yin, Zetong Lin, Huaqiang Wen, Shuai Zhang, Chuanyun Xiao and Semiu Folaniyi Bello designed and performed most of the experiments. Genghua Chen, Zetong Lin, Yunqian Yin, Jiahui Chen and Wen Luo performed bioinformatics analysis. Wen Luo and Qinghua Nie provided funds. Genghua Chen wrote the manuscript.

## FUNDING INFORMATION

This work was supported by the National Key Research and Development Program of China (2021YFD1300102 and 2022YFF1000201), National Scientific Foundation of China (31972544), Science and Technology Program of Guangdong Province (2020B1212060060), China Agriculture Research System of MOF and MARA (CARS‐41) and Local Innovative and Research Teams Project of Guangdong Province (2019BT02N630).

## CONFLICT OF INTEREST STATEMENT

The authors declare no conflicts of interest.

## Supporting information


**DATASET EV 1.** Primer used in this study.

**DATASET EV 2.** A comprehensive long‐read transcriptome of myogenic differentiation constructed by SQANTI3.

**DATASET EV 3.** Alternative splicing events of PM, GM and DM stages detected by SUPPA.

**DATASET EV 4.** Differentially expressed genes of scRNA‐seq between cell types in PM, GM and DM stages.

**DATASET EV 5.** Differentially expressed genes of scRNA‐seq between cell types in muscle cell subclusters.

**DATASET EV 6.** Splice junction usage of comparison between muscle cell and other cell types.

**DATASET EV 7.** Number of differential spliced SJ between cell subpopulations in muscle cells.

**DATASET EV 8.** The detailed information of spliced junction usage between cell subclusters in muscle cells.

**DATASET EV 9.** RIP‐seq of myoblasts transfected into TRA2B‐L or TRA2B‐S.

**DATASET EV 10.** Prediction of the binding motifs for RNA binding proteins.

**DATA S1.** Supporting Information.

## Data Availability

Complete data sets and cloning details are available from the corresponding author on reasonable request. Raw data in this study have been deposited in the Genome Sequence Archive (Genomics, Proteomics & Bioinformatics 2021) in the National Genomics Data Center, China National Center for Bioinformation/Beijing Institute of Genomics, Chinese Academy of Sciences (GSA: CRA008970) that are publicly accessible at https://ngdc.cncb.ac.cn/gsa.^89^
