# Supplementary material for: Bulk and single‐cell alternative splicing analyses reveal roles of TRA2B in myogenic differentiation
Source: Cell Prolif. 2023 Sep 13;57(2):e13545. doi: 10.1111/cpr.13545 (PMC10849790; doi:10.1111/cpr.13545)
Supplement: Supplementary file 11 — DATA S1. Supporting Information. [file CPR-57-e13545-s007.pdf]

Bulk and single cell alternative splicing analyses reveal TRA2B regulates myogenic differentiation by orchestrating target RNAs splicing

Genghua Chen[1, 2], Jiahui Chen[1, 2], Lin-Qi[1, 2], Yunqian Yin[1, 2], Zetong Lin[1, 2], Huaqiang Wen[1, 2], Shuai Zhang[1, 2], Chuanyun Xiao[3], Li Meng[4], Semiu Folaniyi Bello[1,2], Xiquan Zhang[1, 2], Qinghua Nie[1, 2, *], Wen Luo[1, 2, *]

1 College of Animal Science, South China Agricultural University, Guangzhou, Guangdong 510642, China

2 Guangdong Provincial Key Lab of Agro-Animal Genomics and Molecular Breeding, Lingnan Guangdong Laboratory of Modern Agriculture & State Key Laboratory for Conservation and Utilization of Subtropical Agro-bioresources, Key Laboratory of Chicken Genetics, Breeding and Reproduction, Ministry of Agriculture, Guangzhou, Guangdong 510642, China.

3 Department of Microbiology, The Chinese University of Hong Kong, Shatin, N.T., Hong Kong SAR, China.

4 Human and Animal Physiology, Wageningen University, De Elst 1, 6708 WD Wageningen, The Netherlands.

* To whom correspondence should be addressed. Tel: + 86 20 8528 5702; Fax: + 86 20 8528 0740; Email: luowen729@scau.edu.cn (Wen Luo); nqinghua@scau.edu.cn (Qinghua Nie)

Running title: TRA2B regulates myogenesis through alternative splicing

Corresponding author: Wen Luo

Address: College of Animal Science, South China Agricultural University, Guangzhou 510642, Guangdong Province, China

E-mail: luowen729@scau.edu.cn

Corresponding author: Qinghua Nie

Address: College of Animal Science, South China Agricultural University, Guangzhou 510642, Guangdong Province, China

E-mail: nqinghua@scau.edu.cn

Supplementary Materials and methods

10X genomics single-cell library preparation

The suspensions of single cells obtained from in vitro culture were processed using the droplet-based single-cell capture platform, the Chromium controller, from 10x Genomics. Following calculation of accurate volumes using the 'Cell Suspension Volume Calculator Table', the chips were loaded. We aimed to recover approximately 10,000 cells, starting from an initial single-cell suspension concentration of 1000 cells/μl. As individual cells flowed through the microfluidic chip, they were lysed and labeled with a bead containing unique molecular identifiers before being encapsulated in an oil droplet. The resulting emulsion was then subjected to reverse transcription amplification, and the library was prepared according to the instructions provided in the 10x Genomics 3' V3 manual.

Table S1 Classification of long-read RNA-seq

Category	PM	GM	DM	Total/Average
Subreads numbers	3268193	7606964	6885085	17760242/5920081
Average subreads length	2911	1666	1560	-/2046
CCS	276937	684848	514795	1476580/492194
5'-primer transcripts	252619	452017	429995	1134631/378211
3'-primer transcripts	258083	539300	454942	1252325/417442
Ploy (A) transcripts	249346	527828	430060	1207234/402412
Full-length reads	227224	362333	360475	950032/316678
Full-length non-chimeric reads (Flnc)	224659	353637	355322	933618/311206
Average Flnc reads length	3409	2274	2293	-/2659
Polished consensus reads	101172	187567	172755	461494/153832
Average consensus reads length	3626	2534	2553	-/2905
Corrected transcripts	101172	187567	172755	461494/153832
Average length of corrected transcripts	3671	2629	2592	-/2964

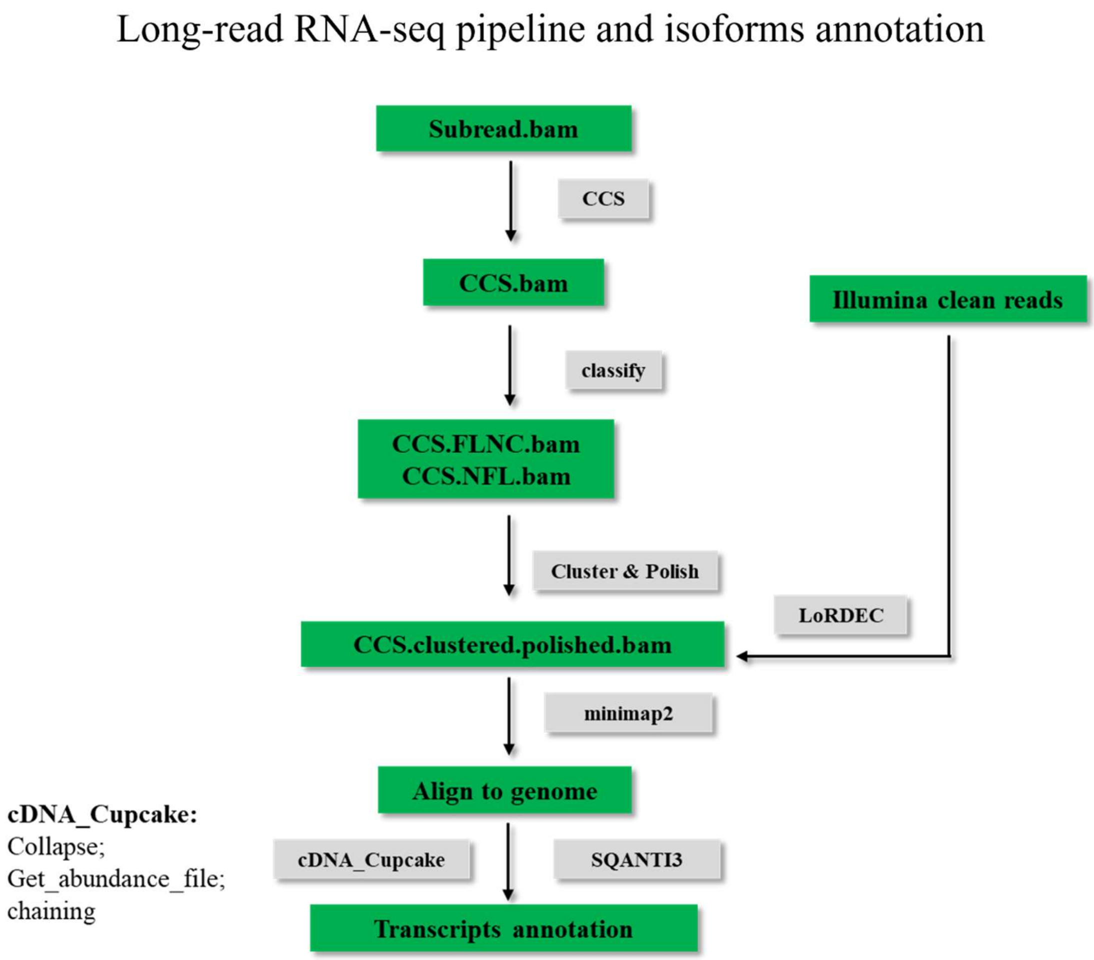

Figure EV1. Long-read RNA-seq and short-read RNA-seq analysis pipeline.

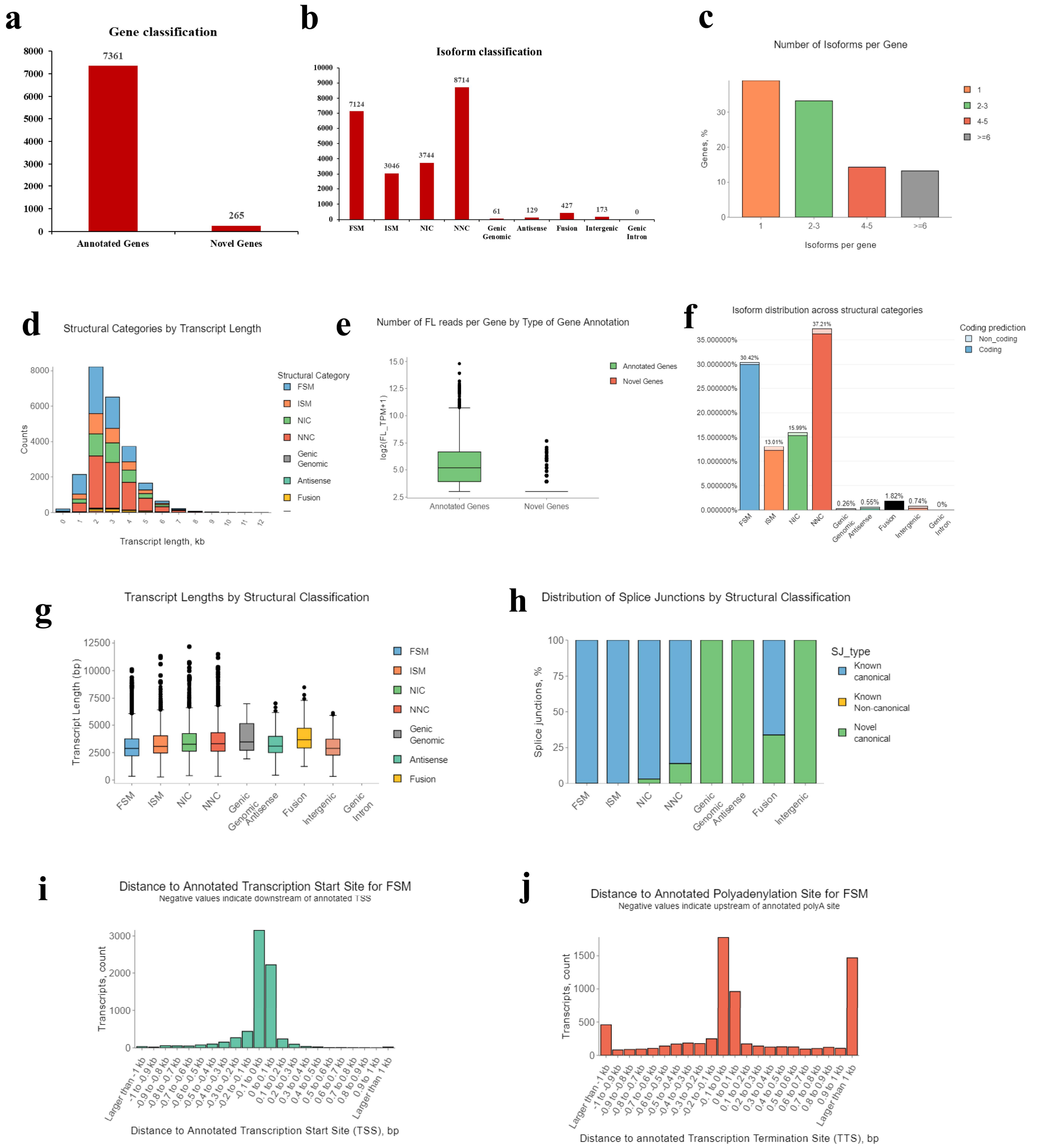

Figure EV 2. An overview of long-read RNA-seq of PM myoblasts. (a) The number of unique genes and isoforms from long-read RNA-seq of PM myoblasts. (b) In-depth characterization of isoforms using SQANTI3 based on isoform category. All isoforms were grouped into nine categories by SQANTI3. FSM, full-splice match; ISM, incomplete splice match; NIC, novel in catalog; NNC, novel not in catalog. (c) The number of isoforms per gene. (d) The distribution of transcript length of structural category. (e) The number of full-length reads for annotated and novel genes. (f) The isoforms distribution characterized by SQANTI3. (g) The length of isoforms characterized by SQANTI3. (h) The distribution of slice junction characterized by SQANTI3. (i) The distance to annotated transcription start site for full splice matched isoforms. (j) The distance to annotated

polyadenylation site for full splice matched isoforms.

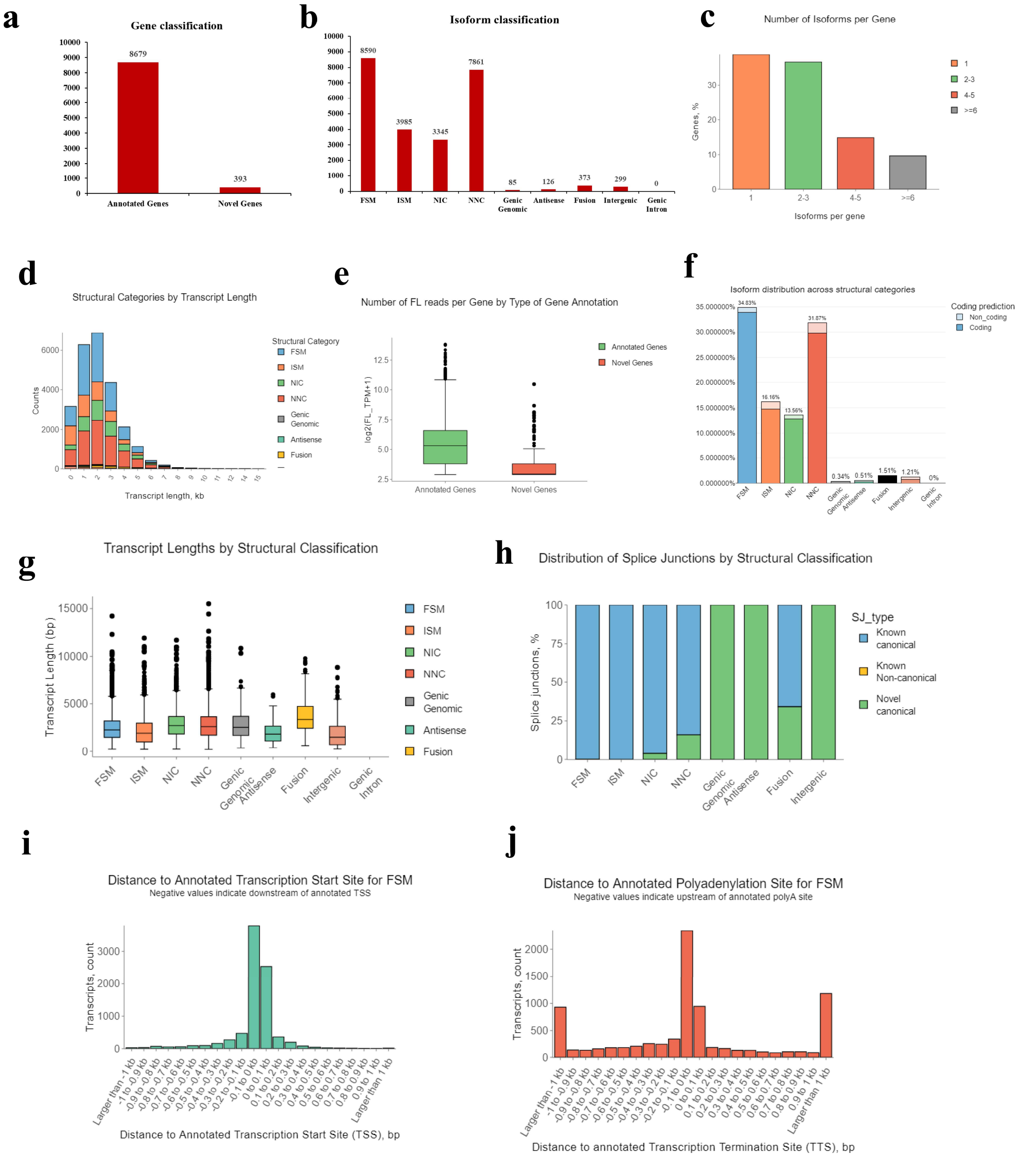

Figure EV 3. An overview of long-read RNA-seq of GM myoblasts. (a) The number of unique genes and isoforms from long-read RNA-seq of GM myoblasts. (b) In-depth characterization of isoforms using SQANTI3 based on isoform category. All isoforms were grouped into nine categories by SQANTI3. FSM, full-splice match; ISM, incomplete splice match; NIC, novel in catalog; NNC, novel not in catalog. (c) The number of isoforms per gene. (d) The distribution of transcript length of structural category. (e) The number of full-length reads for annotated and novel genes. (f) The isoforms distribution characterized by SQANTI3. (g) The length of isoforms characterized by SQANTI3. (h) The distribution of slice junction characterized by SQANTI3. (i) The distance to annotated transcription start site for full splice matched isoforms. (j) The distance to annotated

polyadenylation site for full splice matched isoforms.

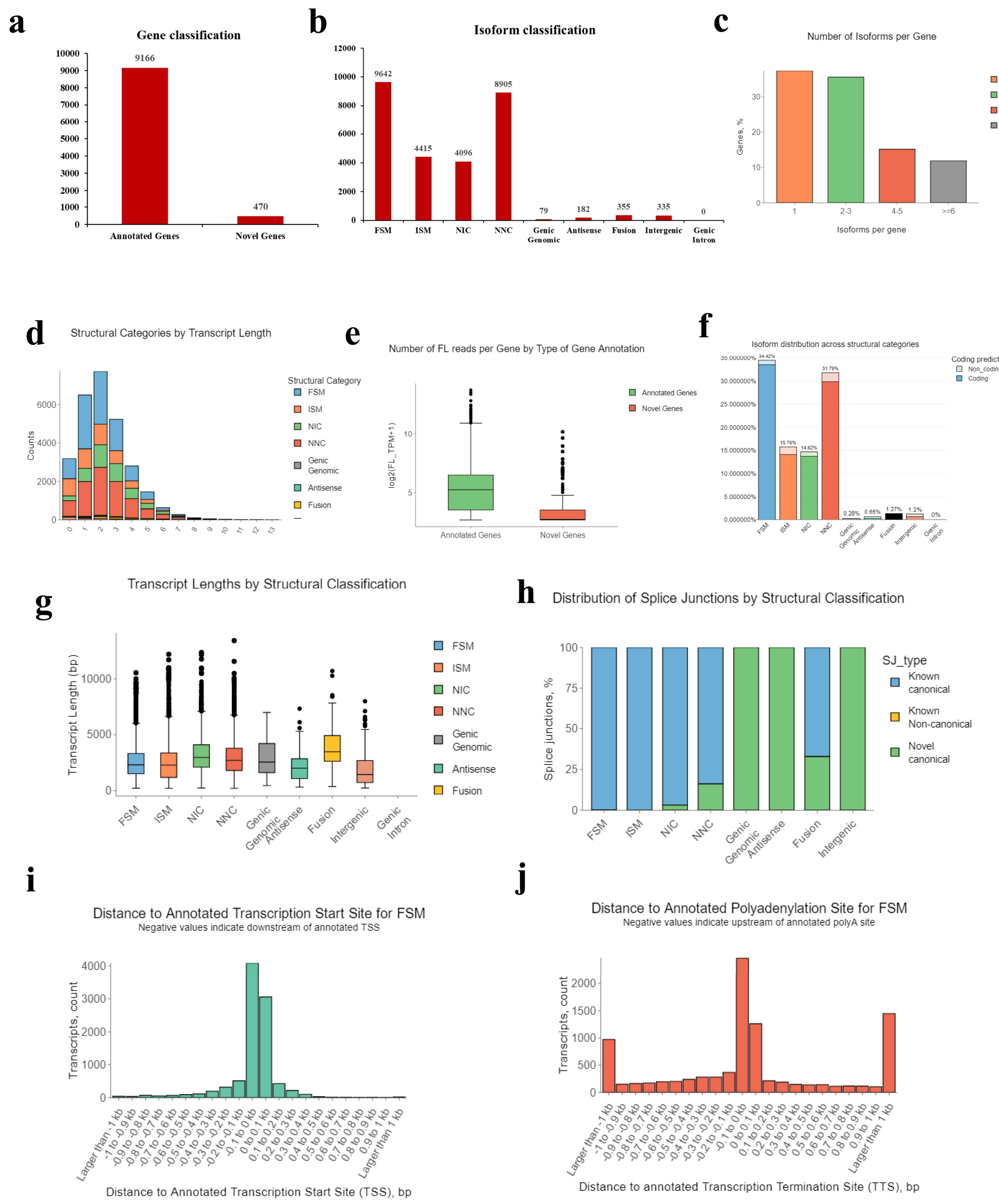

Figure EV 4. An overview of long-read RNA-seq of DM myoblasts. (a) The number of unique genes and isoforms from long-read RNA-seq of DM myoblasts. (b) In-depth characterization of isoforms using SQANTI3 based on isoform category. All isoforms were grouped into nine categories by SQANTI3. FSM, full-splice match; ISM, incomplete splice match; NIC, novel in catalog; NNC, novel not in catalog. (c) The number of isoforms per gene. (d) The distribution of transcript length of structural category. (e) The number of full-length reads for annotated and novel genes. (f) The isoforms distribution characterized by SQANTI3. (g) The length of isoforms characterized by

SQANTI3. (h) The distribution of slice junction characterized by SQANTI3. (i) The distance to annotated transcription start site for full splice matched isoforms. (j) The distance to annotated polyadenylation site for full splice matched isoforms.

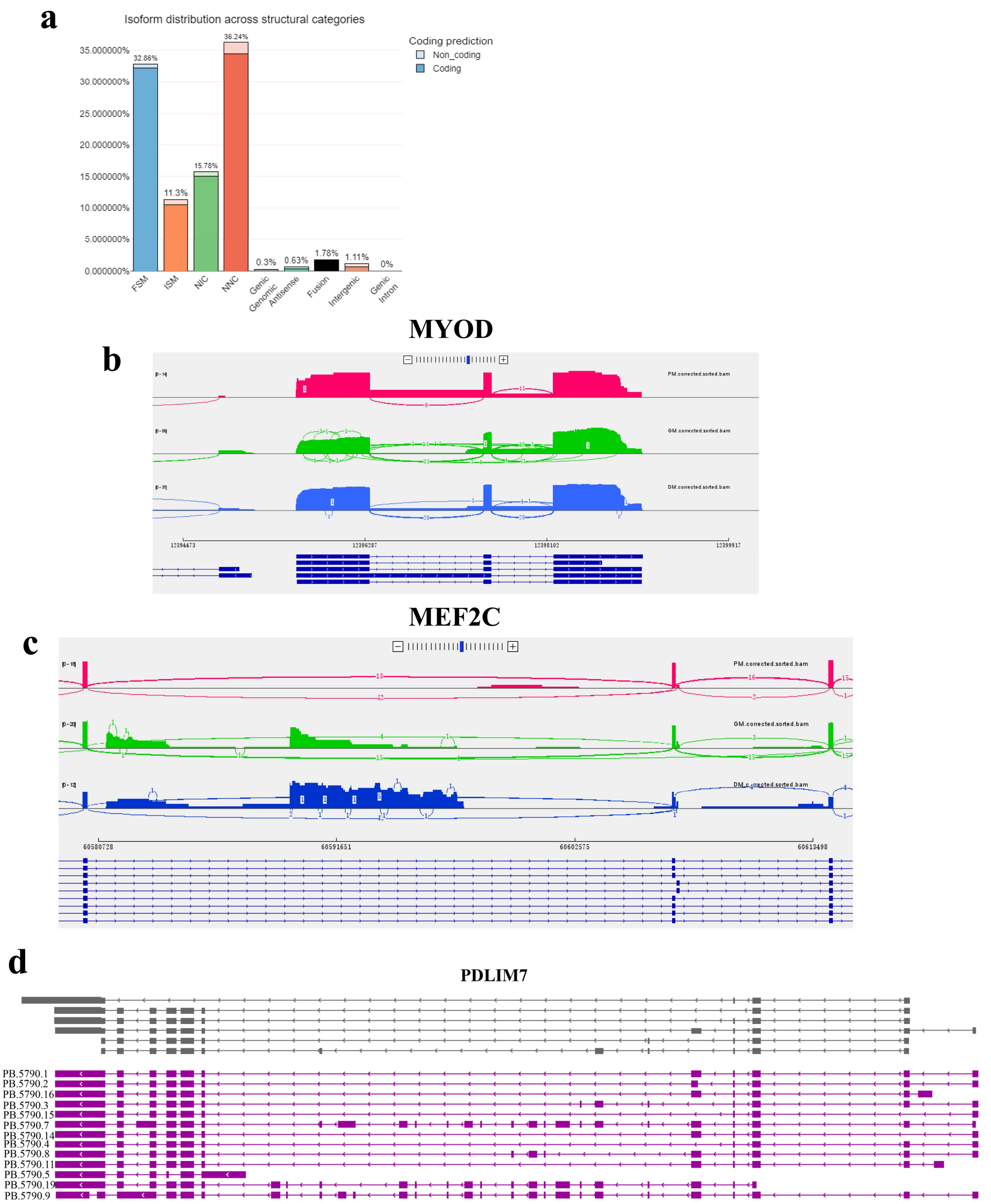

Figure EV 5. An overview of myogenic master transcriptome constructed by PM, GM, and DM long-read RNA-seq. (a) The isoform distribution of the master transcriptome characterized by SQANTI3. (b) A sashimiplot of MyoD1 from master transcriptome. (c) A sashimiplot of MEF2C from master transcriptome. (d) Visualization of full-length isoforms for PDLIM7 mapped to master transcriptome constructed by PM, GM, and DM long-read transcriptome.

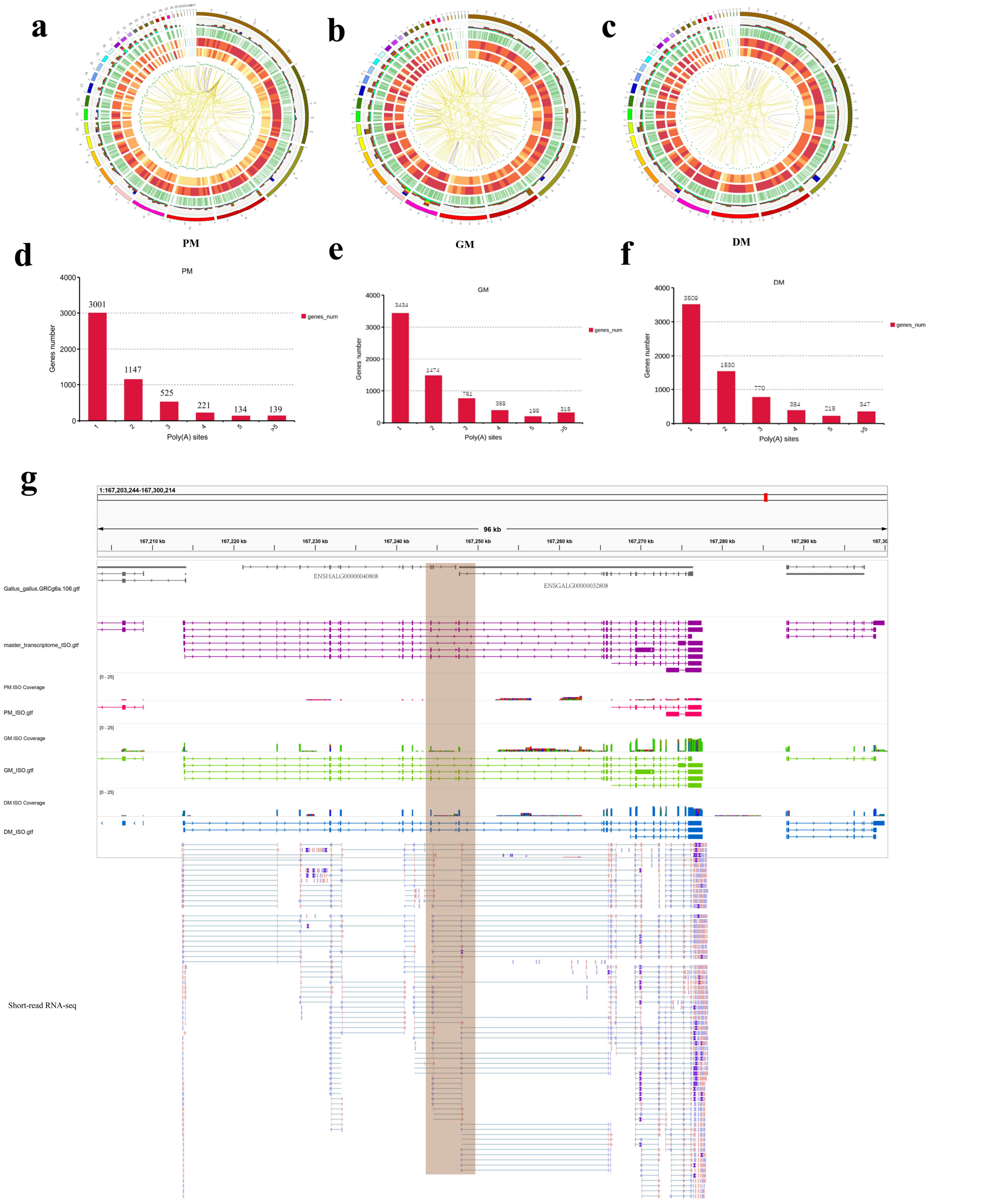

Figure EV 6. (a-c) CIRCOS visualization of fusion transcripts of PM, GM, and DM myoblasts at the genome-wide level. (d-f) The distribution of alternative polyA sites of PM, GM and DM myoblasts. (g) Combined analysis of transcript tracks from long-read RNA-seq and short-read RNA-seq.

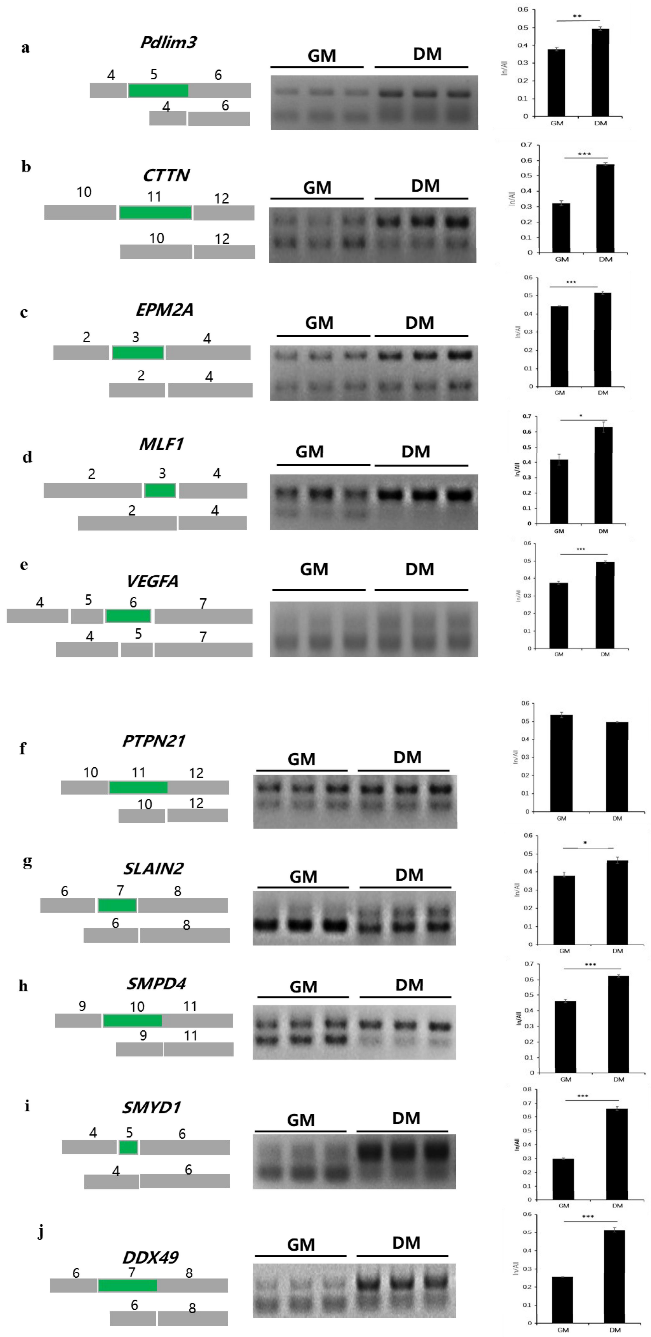

Figure EV 7. RT-PCR of alternative splicing events of indicated genes. The alternative exon was colored with green box. Gray analysis was calculated. All the values are mean ± SEM. The significance of each detected change was evaluated by Student's t-test. *P < 0.05, **P < 0.01, ***P < 0.001.

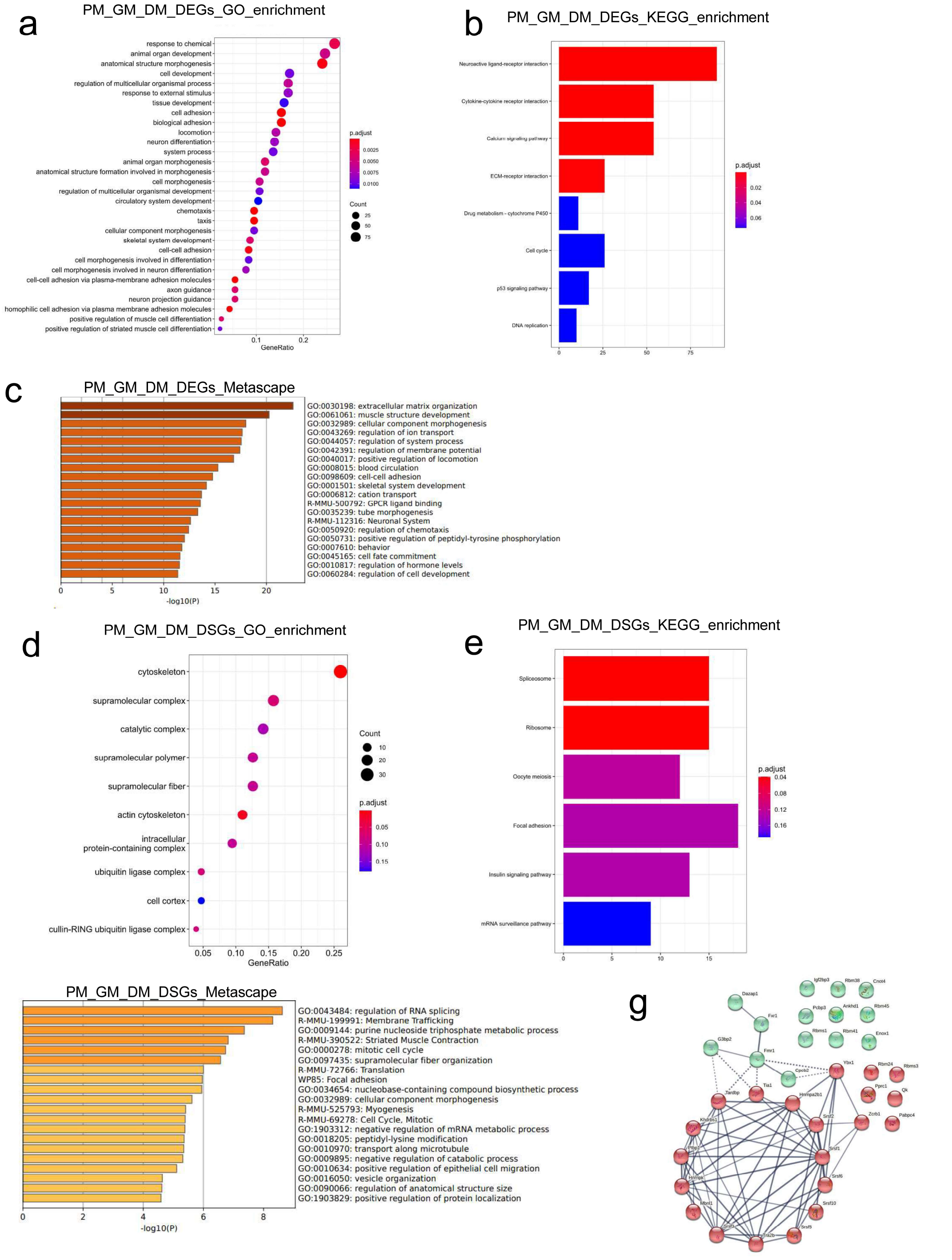

Figure EV 8. Functional enrichment analysis of differentially expressed genes and differentially spliced genes. (a) The GO enrichment of DEGs between PM, GM, and DM myoblasts. (b) The KEGG enrichment of DEGs between PM, GM, and DM myoblasts. (c) The metascape analysis of DEGs between PM, GM, and DM myoblasts. (d) The GO enrichment of DSGs between PM, GM and DM myoblasts. (e) The KEGG enrichment of DSGs between PM, GM and DM myoblasts. (f) The metascape analysis of DEGs between PM, GM, and DM myoblasts. (g) The protein interaction

of RNA binding proteins differentially expressed during chicken myoblast differentiation.

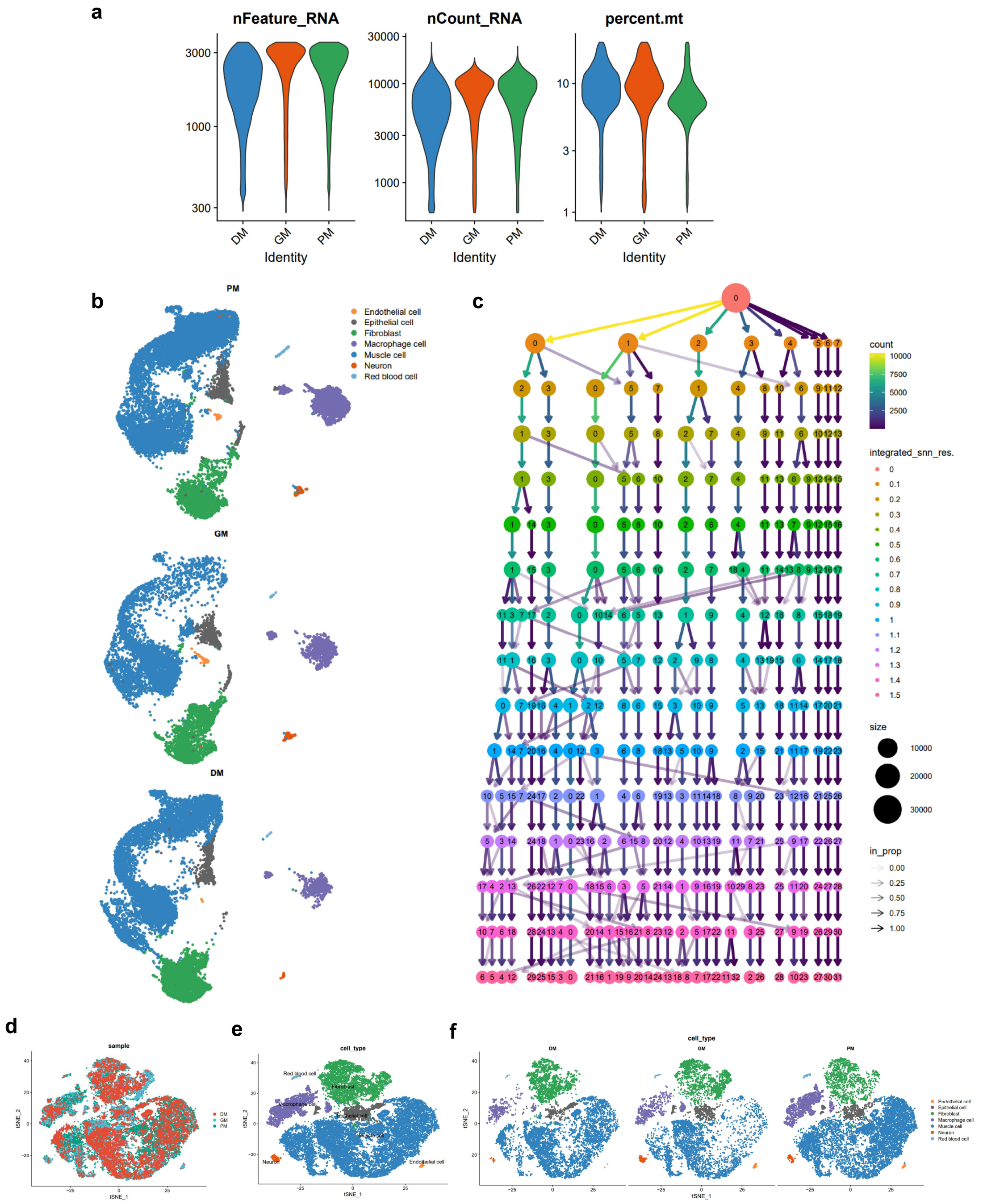

Figure EV 9. Quality control. (a) Number of Unique Molecular Identifiers (UMIs) and genes per cell (nFeature), and percentage of mitochondrial reads. (b) UMAP plots for each stage. (c) Clustree of cell clusters with various resolution. (d) tSNE plot for three stages. (e) tSNE plot showing the distribution of each cell type. (f) tSNE plots showing the distribution of each cell type for three stages.

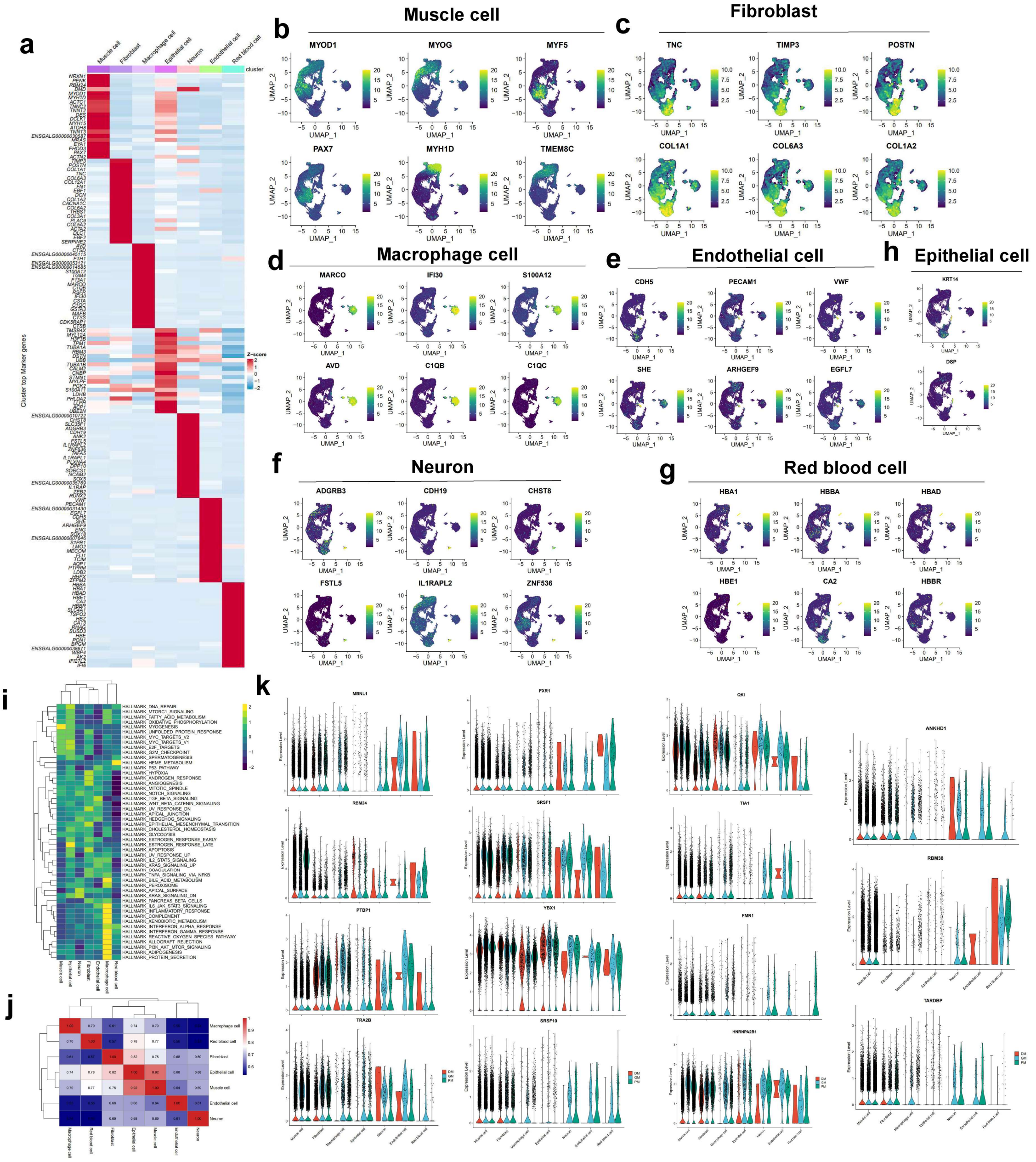

Figure EV 10. Single-cell RNA-seq reveals differential expressed genes and enriched functional processes for major cell cluster during myogenesis. (a) Heatmap of the top 20 genes significantly expressed in each cell types. The differentially expressed genes were identified using the threshold with min.pct = 0.25 and logfc.threshold = 0.25. The full gene list is shown in Supplementary data 6. (b-h) Expression of marker genes for each cell type projected onto the umap plot. Colour intensity refers to the expression level. The yellow indicates high expression and dark blue indicates low expression. (i) GSVA analysis reveal the enriched functional pathway of each cell type. (j) Correlation heatmap between cell types. (k) Violin plots for marker genes of each cell type between PM, GM and DM stages.

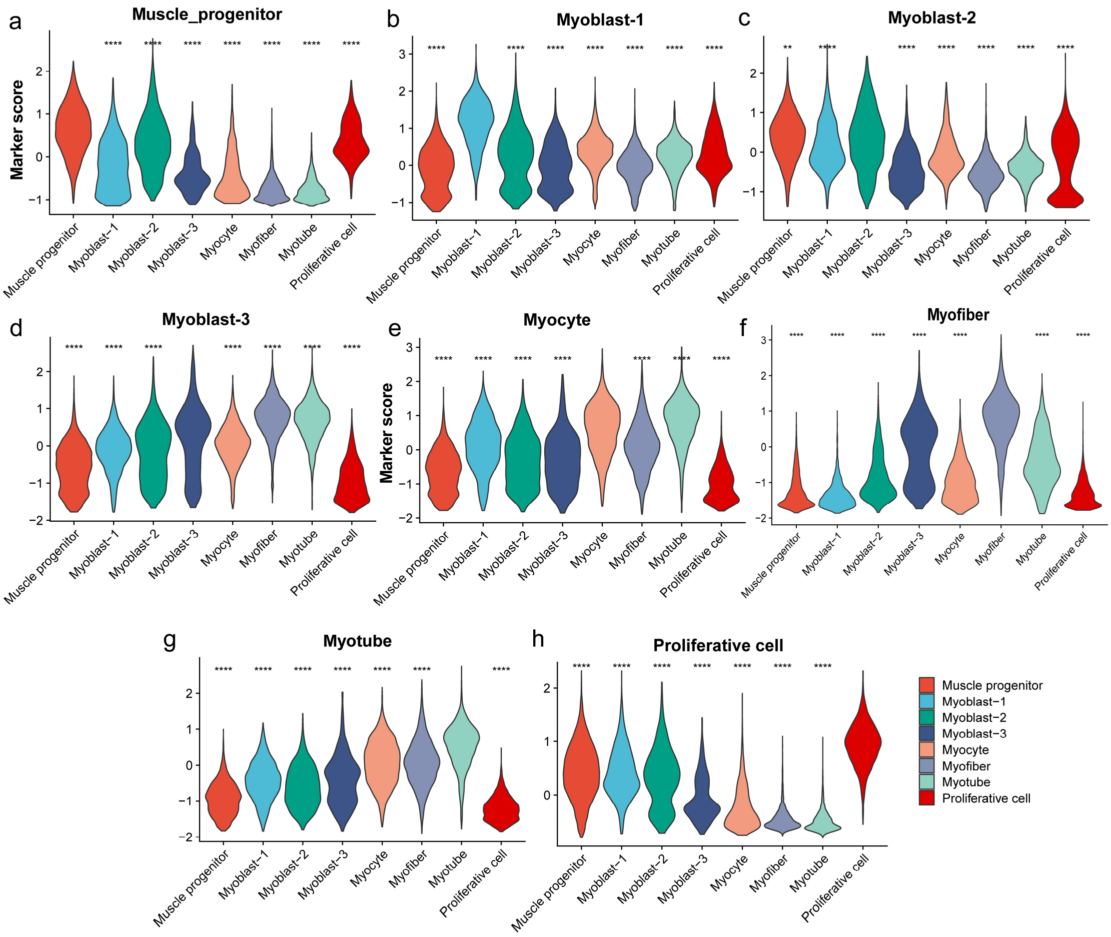

Figure EV 11. Difference in expression of marker signature in subcluster of muscle cell. The marker scores were calculated by AddModuleScore function of Seurat package. The AddModuleScore calculates the average expression of the given marker genes per cell and subtracts it from the average expression of randomly selected control feature. Two-sides Wilcox rank sum test was used for statistical analysis.

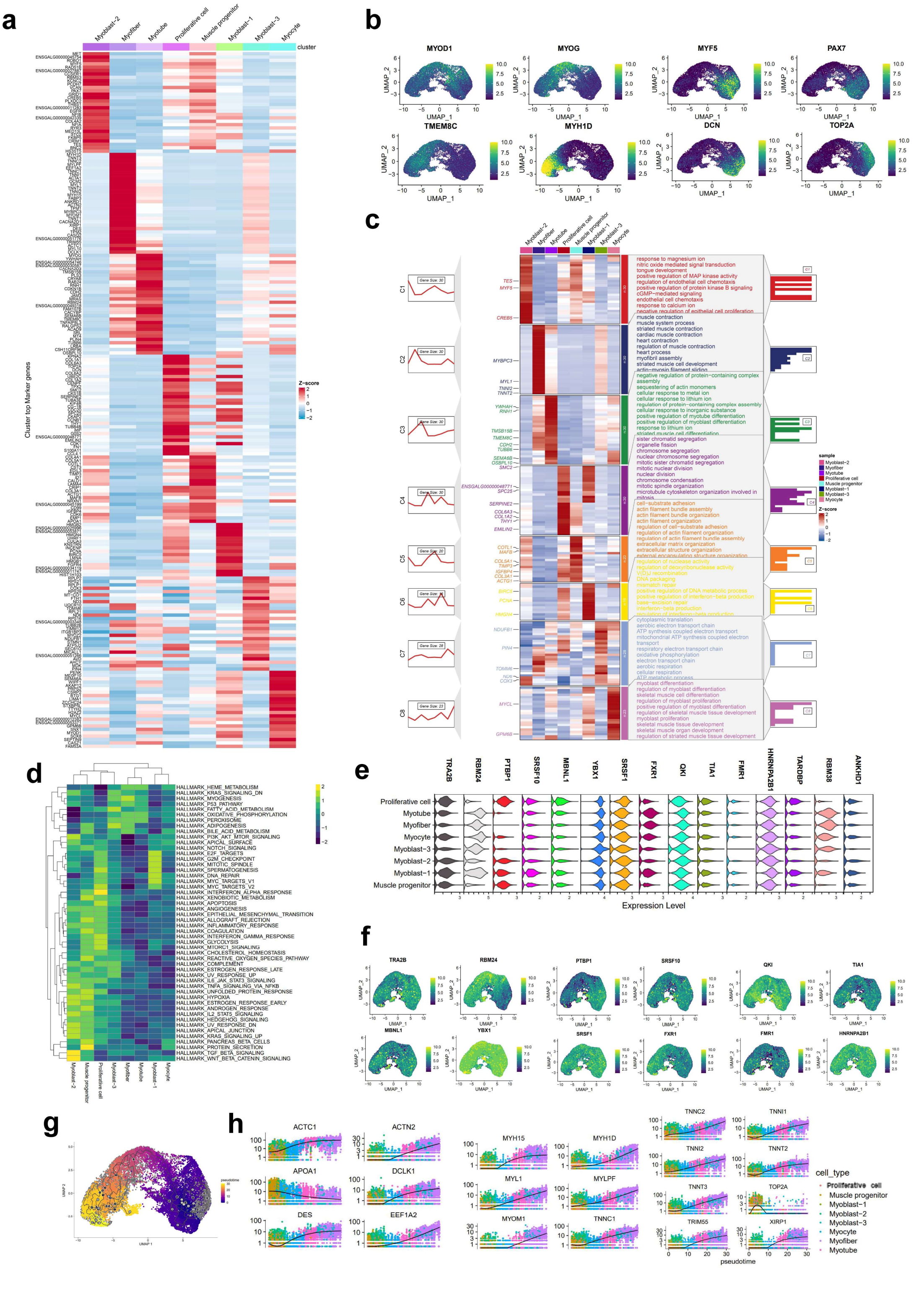

Figure EV 12. Dynamic analysis of gene expression reveals heterogeneity and differentiation trajectory of muscle cell populations. (a) Heatmap of the top 30 genes significantly expressed in each cell types. The differentially expressed genes were identified using the threshold with min.pct = 0.25 and logfc.threshold = 0.25. The full gene list is shown in Supplementary data 7. (b) Expression of marker genes for each cell type projected onto the umap plot. Colour intensity refers

to the expression level. The yellow indicates high expression and dark blue indicates low expression. (c) Biological processes enrichment analysis for differently expressed genes of each cell type. (d) GSVA analysis reveal the enriched functional pathway of each cell type. (e) Violin plots showing the expression of muscle-enriched RBPs between cell types. (f) Expression of muscle-enriched RBPs for each cell type projected onto the umap plot. Colour intensity refers to the expression level. (g) Differentiation trajectory of muscle cells revealed by Monocle. (h) Dotplots showing the expression change of differentially expressed genes in pseudotime order.

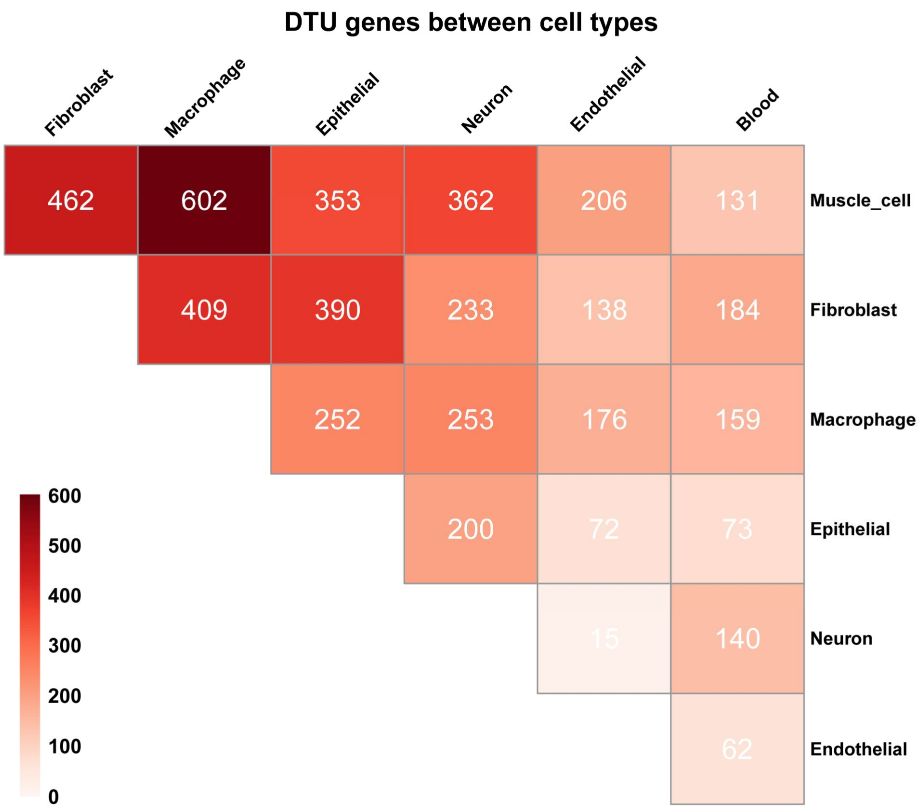

Figure EV 13. The number of genes possessed differential usage transcript between cell types.

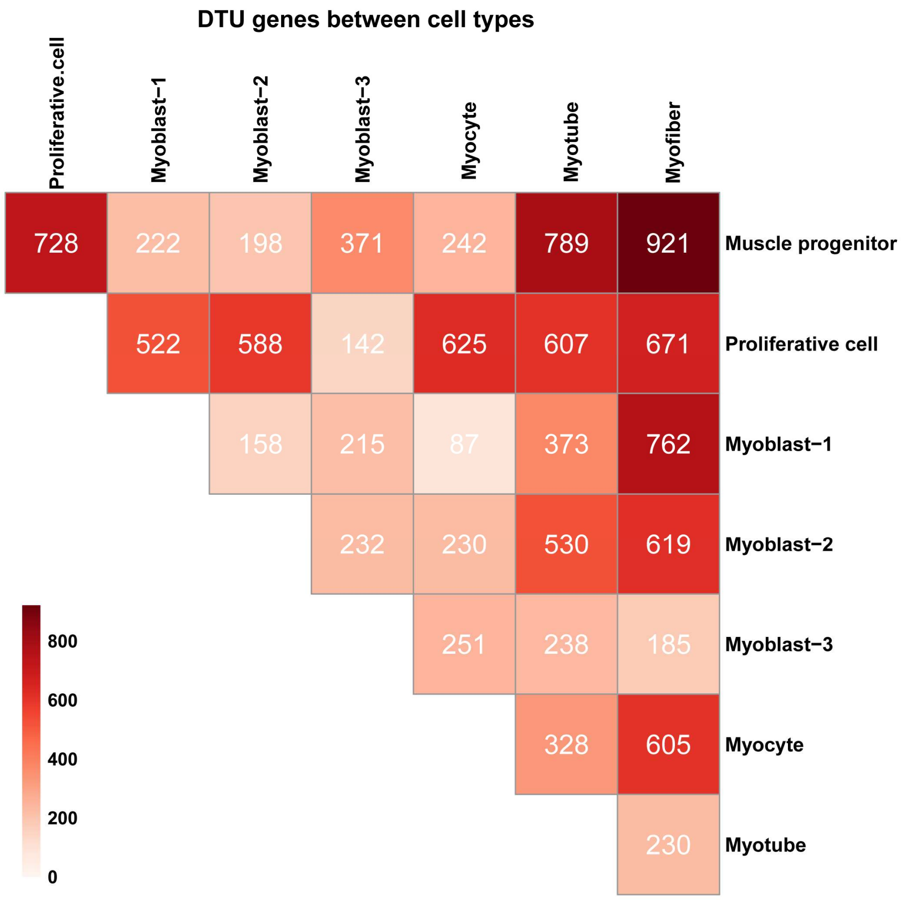

Figure EV 14. The number of genes possessed differential usage transcript between muscle cell types.

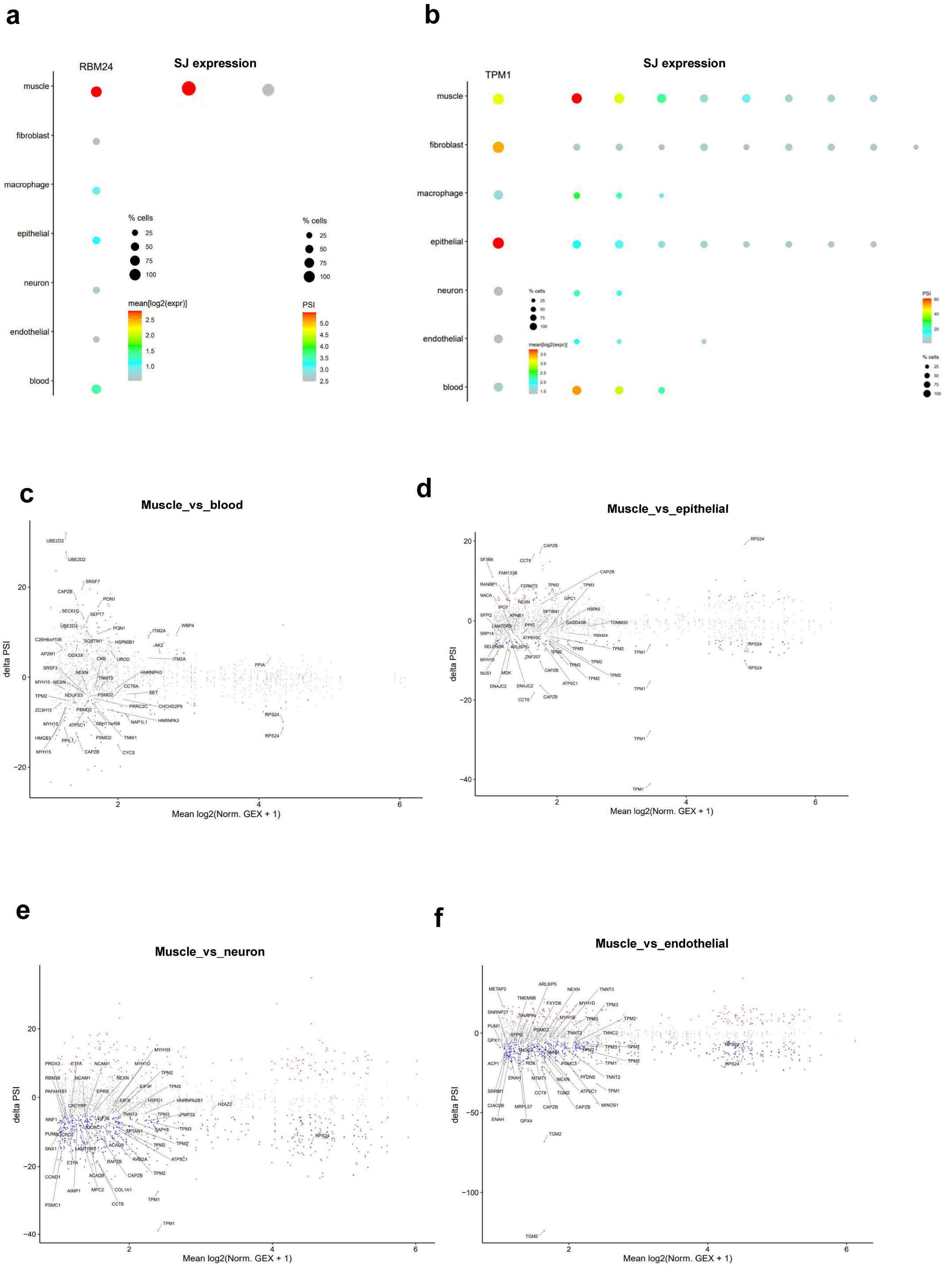

Figure EV 15. Changes in splice junction usage in muscle populations. RBM24 (a) and TPM1 (b) gene expression and splice junction usage across cell types. The left panel indicates the gene expression, while the splice junction usage was shown in right panel. The SJ was arranged by expression level. (c-f) Volcano plot of mean PSI changes in splice junction usage between muscle and other cell types. X-axis indicates the mean normalized gene expression across two cell

populations, while the Y-axis indicates the change in mean PSI values. The full list of gene expression and PSI value is in Supplementary data 8.

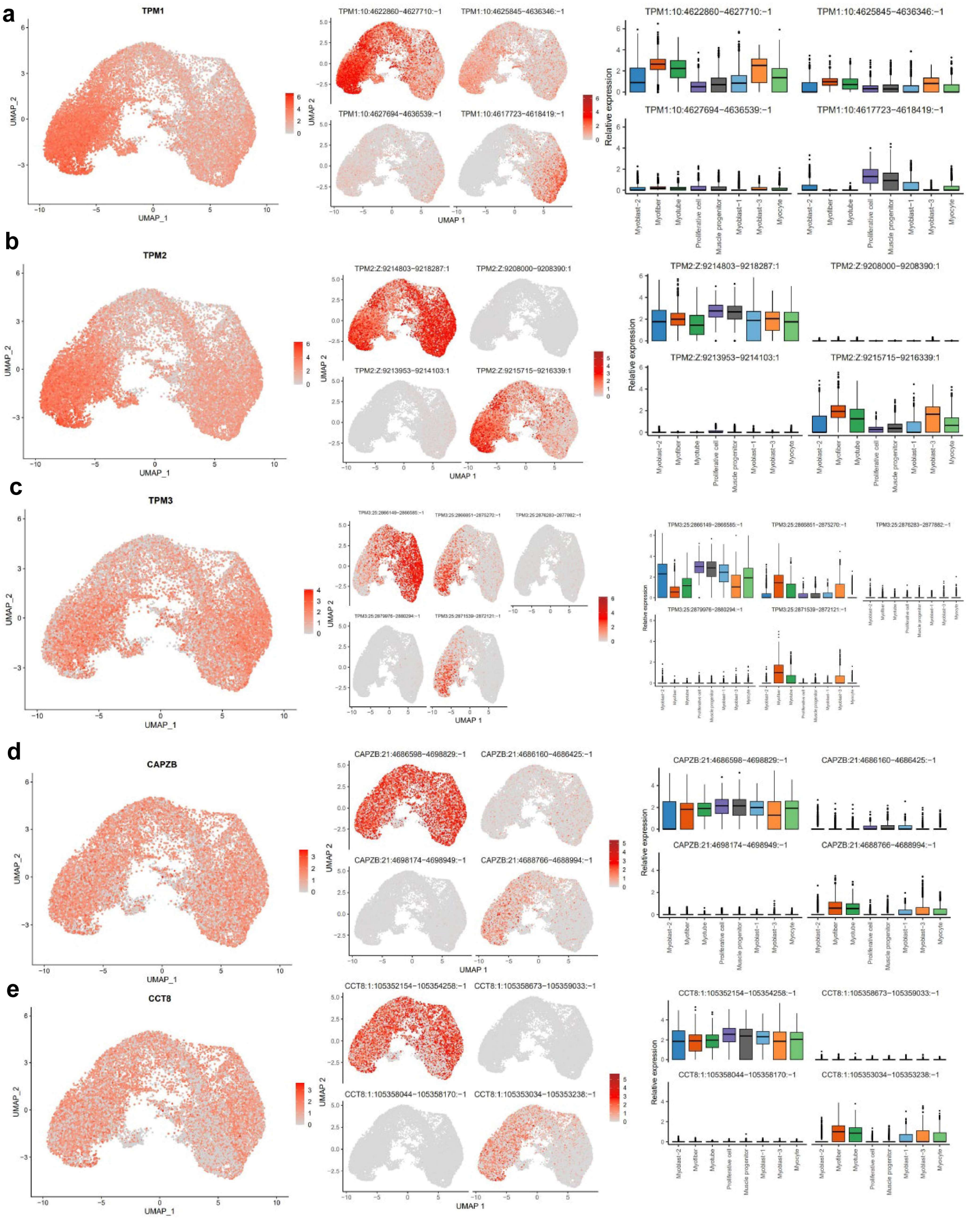

Figure EV 16. Differential transcript usage across muscle cell populations detected by Sierra. Left panel: gene expression of indicated genes visualized on umap plot; Middle panel: relative peaks expression for indicated genes projected onto umap plot; right panel: relative expression of peaks between cell populations.

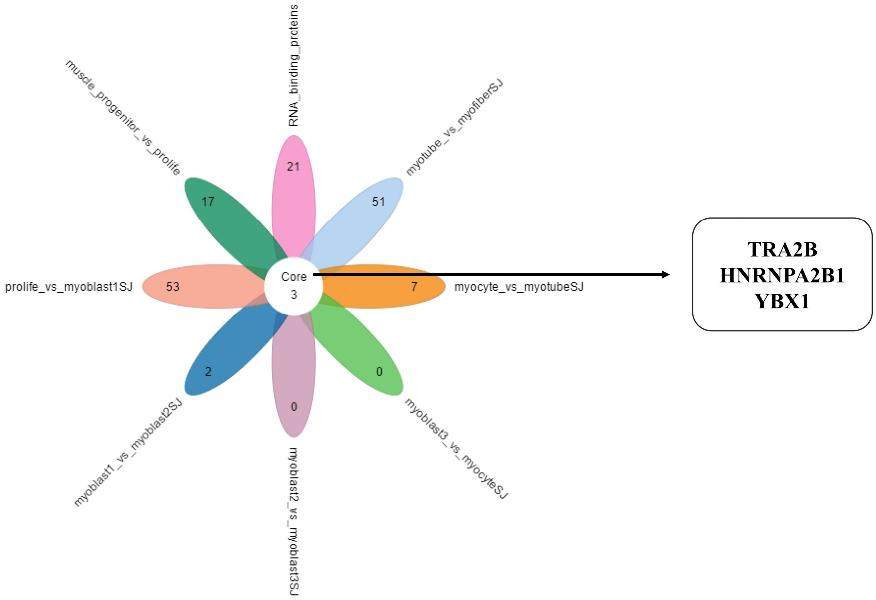

Figure EV 17. RNA-binding proteins with splice junction expression changes during myogenic differentiation.

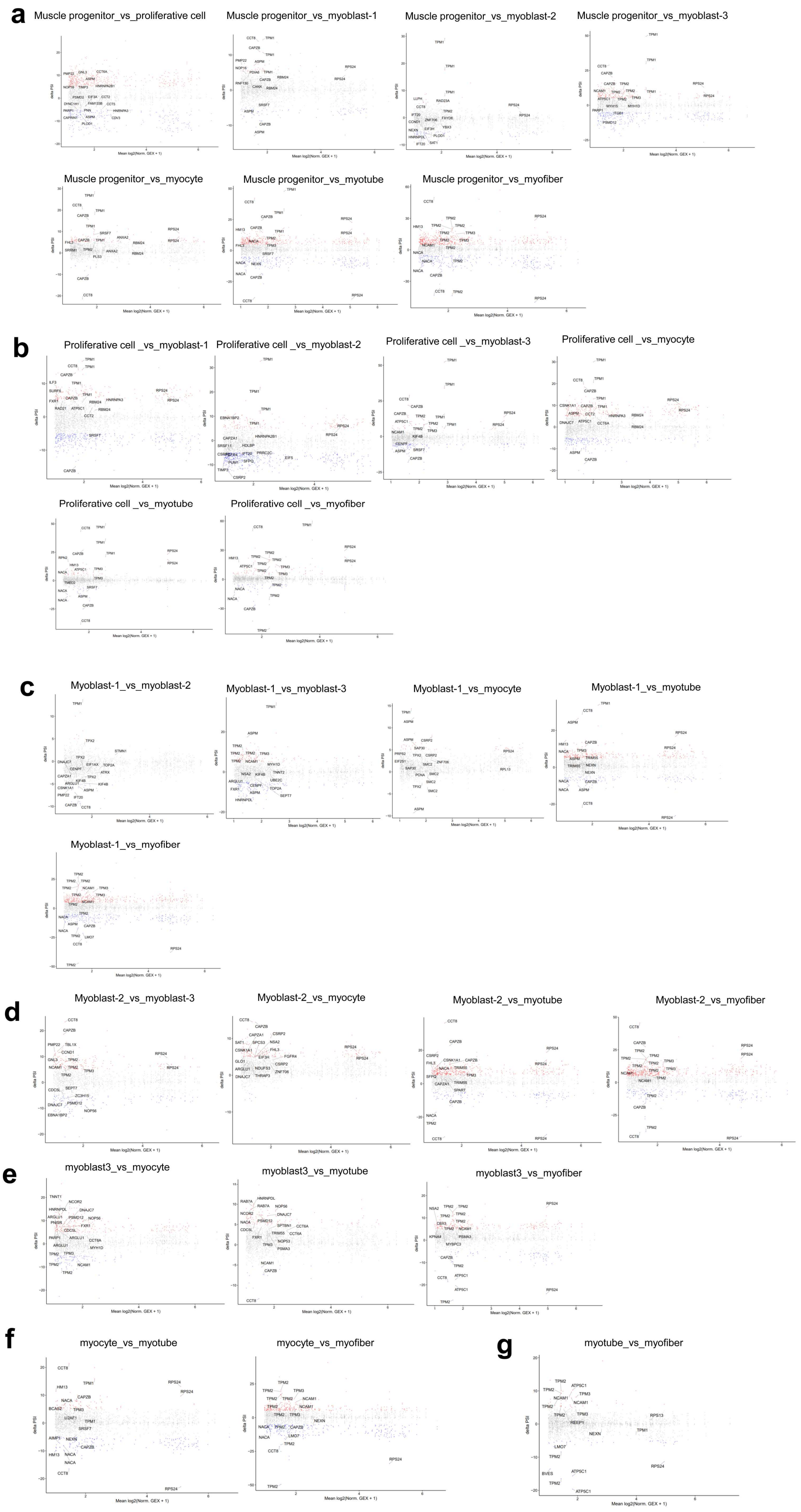
a
Muscle progenitor_vs_proliferative cell
Muscle progenitor_vs_myoblast-1
Muscle progenitor_vs_myoblast-2
Muscle progenitor_vs_myoblast-3
Muscle progenitor_vs_myocyte
Muscle progenitor_vs_myotube
Muscle progenitor_vs_myofiber
b
Proliferative cell _vs_myoblast-1
Proliferative cell _vs_myoblast-2
Proliferative cell _vs_myoblast-3
Proliferative cell _vs_myocyte
Proliferative cell _vs_myotube
Proliferative cell _vs_myofiber
c
Myoblast-1_vs_myoblast-2
Myoblast-1_vs_myoblast-3
Myoblast-1_vs_myocyte
Myoblast-1_vs_myotube
Myoblast-1_vs_myofiber
d
Myoblast-2_vs_myoblast-3
Myoblast-2_vs_myocyte
Myoblast-2_vs_myotube
Myoblast-2_vs_myofiber
e
myoblast3_vs_myocyte
myoblast3_vs_myotube
myoblast3_vs_myofiber
f
myocyte_vs_myotube
myocyte_vs_myofiber
g
myotube_vs_myofiber

Figure EV 18. Volcano plot of mean PSI changes in splice junction usage between two cell populations. X-axis indicates the mean normalized gene expression across two cell populations, while the Y-axis indicates the change in mean PSI values. The full list of gene expression and PSI value is in Supplementary data 9.

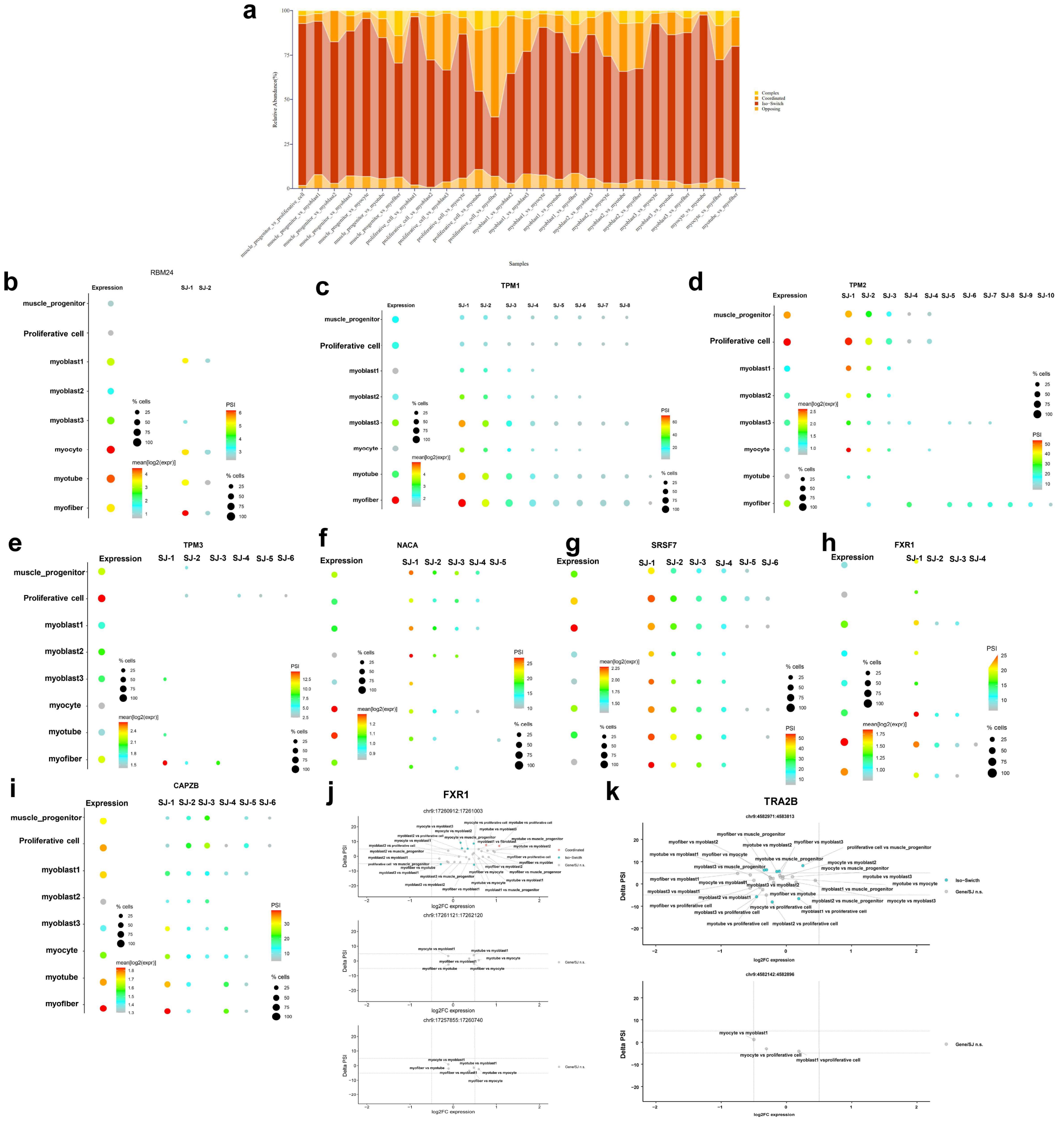

Figure EV 19. Changes in splice junction usage in muscle cell populations based on splice junction. (a) The percentage of gene-splicing dynamics between cell types. Gene-splicing dynamics that integrated gene expression and splice junction usage was classified into coordinated, opposing, isoform-switching (iso-switch) and complex categories. (b-i) gene expression and splice junction usage across indicated cell types. (j-k) Alteration in splice junction usage compared to alteration of gene expression between both cell types.

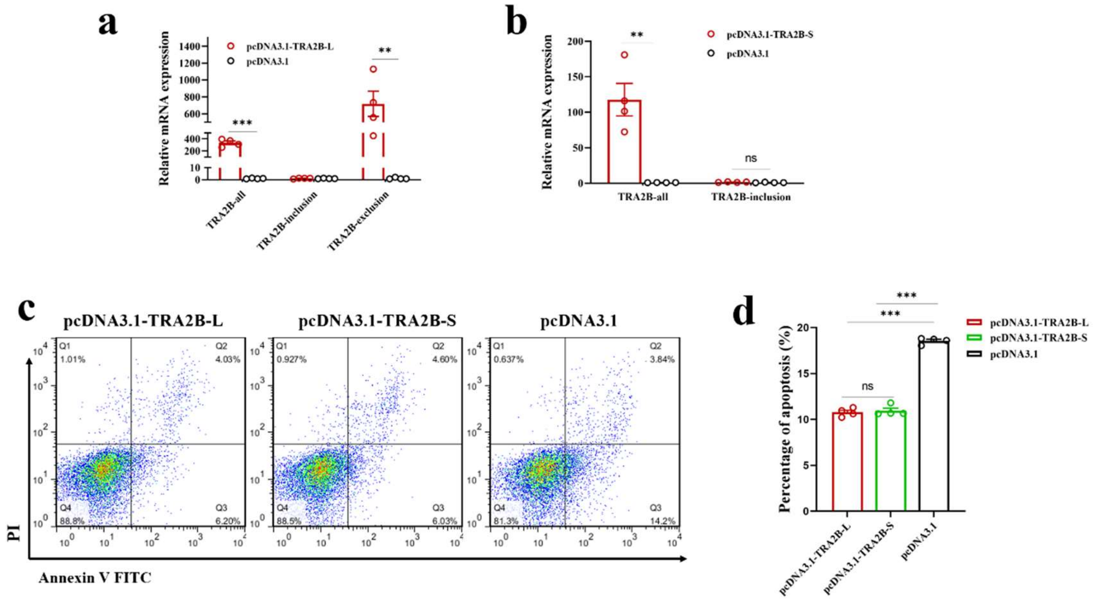

Figure EV 20. (a,b) qPCR analysis of indicated transcripts using specific primers. (c,d) Cell apoptosis was determined by flow cytometry after overexpression of TRA2B and TRA2B-S (n = 4).

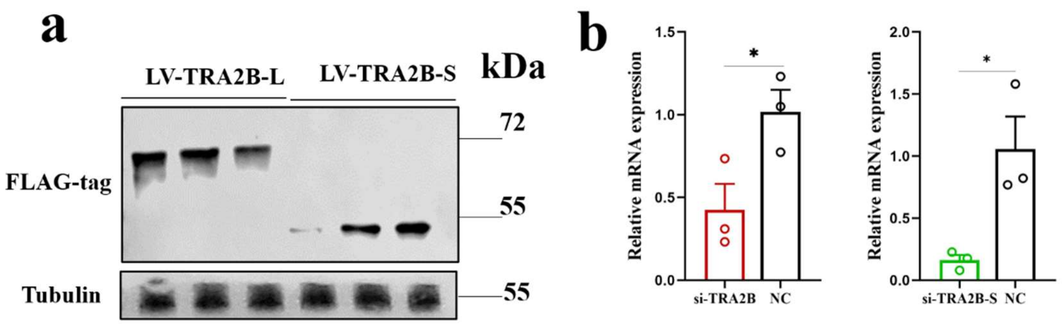

Figure EV 21. TRA2B isoforms expression in vivo. (a) Lentivirus mediated overexpression of TRA2B-L and TRA2B-S in vivo detected by WB. (b) mRNA expression of TRA2B andTRA2B-S after in-vivo siRNA treatment. All the values are mean ± SEM. The significance of each detected change was evaluated by Student's *t*-test. *$P < 0.05$.

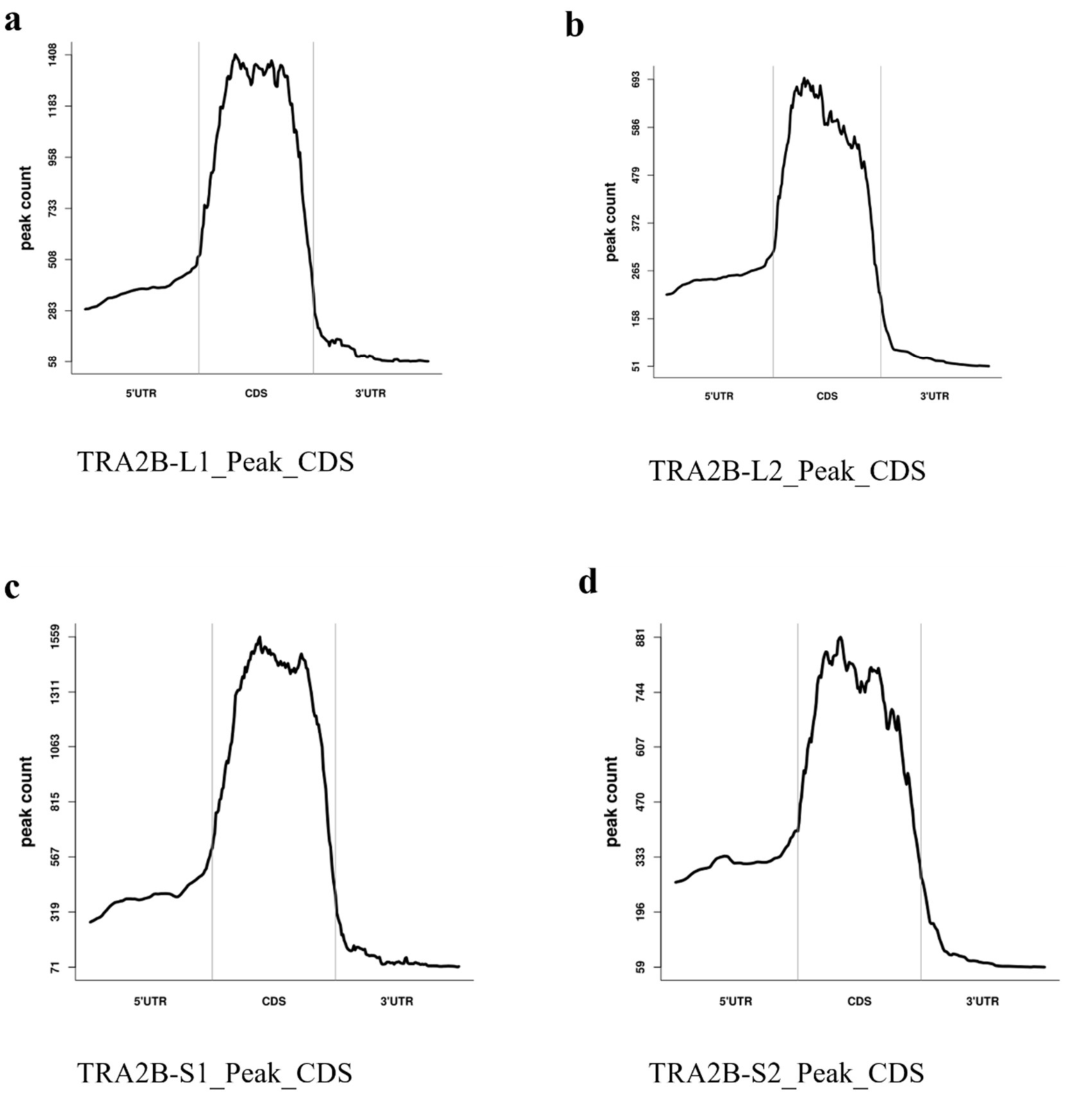

Figure EV 22. The distribution of peak count in gene body.

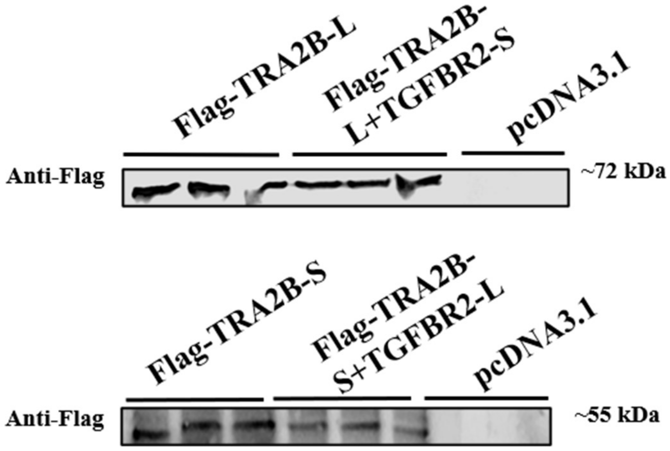

Figure EV 23. Western blot analysis for TRA2B-L and TRA2B-S overexpression.
